# The Role of Genetic Factors in the Differential Invasion Success of Two *Spartina* Species in China

**DOI:** 10.3389/fpls.2022.909429

**Published:** 2022-05-30

**Authors:** Feifei Li, Xiaoyan Liu, Jinfang Zhu, Junsheng Li, Kexiao Gao, Caiyun Zhao

**Affiliations:** State Key Laboratory of Environmental Criteria and Risk Assessment, Chinese Research Academy of Environmental Sciences, Beijing, China

**Keywords:** *Spartina alterniflora*, biological invasions, RAD, genetic paradox, environmental adaptation

## Abstract

Biological invasions have become one of the greatest threats to global biodiversity and ecosystem conservation. Most previous studies have revealed how successful invasive species adapt to new environments and climate change through phenotypic and genetic evolution. Some researchers suggested that understanding unsuccessful or less successful biological invasions might be important for understanding the relationships between invasion adaptability and climate factors. We compared the sexual reproduction ability, genetic diversity, and gene × environment interaction in two intentionally introduced alien species in China (*Spartina anglica* and *Spartina alterniflora*) based on restriction site-associated DNA (RAD) sequencing. After more than 50 years, the distribution of *S. alterniflora* has rapidly expanded, while *S. anglica* has experienced extreme dieback. A total of 212,939 single nucleotide polymorphisms (SNPs) for the two *Spartina* species were used for analysis. The multilocus genotype (MLG) analysis revealed that clonal reproduction was the prevalent mode of reproduction in both species, indicating that a change in the mode of reproduction was not the key factor enabling successful invasion by *Spartina*. All genetic diversity indicators (*He*, *Ho*, π) in *S. alterniflora* populations were at least two times higher than those in *S. anglica* populations, respectively (*p* < 0.001). Furthermore, the population genetic structure and stronger patterns of climate-associated loci provided support for rapid adaptive evolution in the populations of *S. alterniflora* in China. Altogether, our results highlight the importance of genetic diversity and local adaptation, which were driven by multiple source populations, in increasing the invasiveness of *S. alterniflora*.

## Introduction

Biological invasions have become one of the greatest threats to global biodiversity and ecosystem conservation (Clavero and García-Berthou, 2005; Simberloff et al., 2013; Early et al., 2016). Understanding the mechanisms of invasion would contribute to methodologies for its prevention and control. In recent years, many researchers have explored invasion mechanisms by examining the genetic backgrounds of invasive alien species, especially the links between genetic diversity, bottlenecks, founder events, adaptive evolution, and invasion success (Dlugosch and Parker, 2008; Prentis et al., 2008; Estoup et al., 2016). Although the genetic diversity of invasive populations may be reduced due to founder events and genetic bottlenecks, most successful invasive species can overcome these negative effects through multiple introductions, phenotypic plasticity, asexual reproduction and hybridization. For instance, *Ambrosia artemisiifolia* invaded European, Australian and Chinese areas with high levels of genetic diversity due to multiple introductions (Genton et al., 2005; Gaudeul et al., 2011; van Boheemen et al., 2017; Li et al., 2019b). Phenotypic plasticity rather than genetic adaptation helps *Alternanthera philoxeroides* (Geng et al., 2007; Roman and Darling, 2007) and *Spartina densiflora* (Castillo et al., 2018) colonize a wide range of habitats. Some populations of *Eichhornia crassipes* in China can successfully invade without genetic variation via clonal reproduction in the introduced range (Ren et al., 2005; Roman and Darling, 2007). *Spartina anglica* successfully invaded the British Isles (Raybould et al., 1991) through interspecies hybridization. Therefore, disadvantages associated with founder events might be overstated (Estoup et al., 2016), and genetic diversity does not necessarily predict invasion success (Roman and Darling, 2007).

Some researchers hypothesized that understanding unsuccessful or less successful biological invasions is also important for determining the role of genetic diversity and bottlenecks in invasion success (Fridley et al., 2007; Roman and Darling, 2007; Zenni and Nuñez, 2013). Comparing genetic and environmental characteristics between successful and unsuccessful populations can also help reveal mechanisms producing fitness variations in different environments, which are valuable in studies of genotype-by-environment interactions in the introduced range (Lee, 2002). Wellband et al. (2017) showed that allelic diversity reduction was associated with lower invasion success by comparing highly successful and less successful invasive species of gobies and tunicates. Following this study, the authors used functional genetic markers to test the link between genetic selection and the invasion success of two pairs of invasive goby species. They proposed that increased evolutionary potential in invaded ranges may be associated with invasion success (Wellband et al., 2018). However, studies on unsuccessful or less successful invasions of different biological groups remain limited.

*Spartina anglica* and *Spartina alterniflora* are perennial grasses native to British estuaries and the Atlantic and Gulf Coast estuaries of North America, respectively. *S. anglica* originated by natural chromosome doubling of an infertile hybrid (*S*. × *townsendii*) between the European native species *S. maritima* and the alien species *S. alterniflora* (Groves and Groves, 1880). The new fertile dodecaploid species has rapidly expanded in salt marshes in several European countries (Gray and Raybould, 1991). As *S. anglica* and *S. alterniflora* are “ecological engineers,” their histories of introduction in China were recorded in detail. While *S. anglica* populations expanded rapidly on the European continent, they experienced dieback in coastal China. The species was introduced to China from England in 1963 (Chung, 1993), with planting sites as far as Dongow, Liaoning, in the north, and Hepu, Guangxi, in the south and a total planting area of almost 36,000 ha in 1981. *S. alterniflora* was introduced to China later from North Carolina, Georgia, and Florida of North America in 1979 (Chung, 1993). However, after more than 50 years, there were only three sites with *S. anglica* in Jiangsu and Zhejiang in 2000, with the area shrunken to 50 ha, whereas the area of *S. alterniflora* had expanded to 112,000 ha, as reported by An et al. (2007).

Most studies have proposed that the coexistence of various intraspecific hybrids, and mixtures of the three ecotypes sampled from their native ranges is the main reason for the wide spread of *S. alterniflora* (Xia et al., 2015), even though the genetic diversity of populations in China was lower than that in native populations (Deng et al., 2007; Xia et al., 2015; Bernik et al., 2016). Such genetic admixture within or between species in the invaded range may allow them to adapt to local conditions via increases in heterozygote frequency and the production of novel genotypes (Ellstrand and Schierenbeck, 2006; Schierenbeck and Ellstrand, 2008; Rius and Darling, 2014). On the basis of chloroplast sequence analysis and reciprocal transplant experiments, Qiao et al. (2019) reported that genetic admixture has facilitated the evolution of super competitive genotypes of *S. alterniflora* in China and that the super competitive genotypes could overcome the negative correlation between plant height and shoot regeneration in the invasive range. In addition, some studies have indicated that a lack of nitrogen in soil (Li et al., 2007), high plant density (Li et al., 2009), and unsuitable soil texture (Liu et al., 2016a) can cause dieback in *S. anglica*. One important reason for the failure of *S. anglica* in China is weak sexual propagation, while *S. alterniflora* had a strong capacity for both sexual and asexual reproduction (Chung, 1985; Xu and Zhuo, 1985; Xiao et al., 2011). Li et al. (2008) studied the breeding system of the species in one field site and a greenhouse, and the results implied that protogyny, poor pollen quality, and abnormal pollen grains and pollen tubes were the main causes of low seed production in *S. anglica* in coastal China. However, no studies have compared the genetic characteristics and genotype-by-environment interactions of the successful invader *S. alterniflora* and the less successful invader *S. anglica*.

Moreover, the distribution areas of *S. anglica* have become unclear in recent years. As reported by An et al. (2007), there were no populations of *Spartina* in Liaoning. Based on geographical information systems (GISs), global positioning systems (GPSs), and on-site investigations in 2007, Zuo et al. (2012) found 1 ha of *S. anglica* in Liaoning, 10 ha in Hebei, 3 ha in Shandong, 1 ha in Jiangsu, and 1 ha in Guangdong. The authors found that populations of *S. alterniflora* were also distributed in these provinces and occupied larger areas than *S. anglica*. Zhang et al. (2017) also reported one population of *S. alterniflora* in Liaoning but no *S. anglica*. In 2019, we found and identified *S. anglica* in Dandong and Huludao cities of Liaoning Province. We chose four populations of *S. alterniflora*, which are distributed from north to south in China, for comparison with the two populations of *S. anglica*. We used restriction site-associated DNA (RAD) sequencing (Baird et al., 2008) to improve the resolution to detect finer-scale spatial patterns of genetic structure and diversity. This sequencing method allows concurrent single nucleotide polymorphism (SNP) identification and genotyping via high-throughput sequencing of flanking regions of restriction enzyme digestion sites dispersed throughout the genome. Here, we addressed three main questions: (1) Did low sexual reproduction cause the dieback of *S. anglica* in coastal China? (2) Does the successful invasive species *S. alterniflora* have higher genetic diversity than the less successful invasive species *S. anglica*? (3) Do the highly successful invasive species *S. alterniflora* and less successful invasive species *S. anglica* differ in their levels of genetic adaptation? We also expected to provide evidence to clarify the role of genetic diversity in the colonization or invasion failure of species.

## Materials and Methods

### Sample Collection and Species Confirmation

We collected leaf samples from 88 individuals of *Spartina* from six sites (14–15 leaf samples per site) along the coastline of China in October 2018 (Supplementary Figure 1). From north to south, the six populations were distributed in Dandong (DD), Huludao (HLD), Tanggu (TG), Cixi (CX), Quanzhou (QZ), and Fangchenggang (FCG). Information on the numbers of individuals and locations of populations is listed in Table 1. Within each population, the distance between each pair of sampled individuals was approximately 10 m. We also obtained three pieces of soil samples from each site, and the depth of the soil samples was 0–25 cm.

**TABLE 1:** Location information, number of sampled individuals, and genotypic diversity in populations of *Spartina anglica* and *Spartina alterniflora*.

Species	Pop	Collection location	Lon. (°E)	Lat. (°N)	*N*	*G*	G/N	*H*	λ	*E*	*I*_*A*_	*r*_*d*_
*Spartina anglica*	DD	Dandong, Liaoning	124.209°	39.872°	15	15	1.000	2.708	0.933	1.000	7.132	0.001
	HLD	Huludao, Liaoning	120.962°	40.799°	15	6	0.400	1.173	0.533	0.512	22.653*	0.005*
	Total	-	-	-	30	21	0.700	2.634	0.867	0.503	32.076*	0.005*
*Spartina alterniflora*	TG	Tanggu, Tianjin	117.713°	38.958°	14	14	1.000	2.639	0.929	1.000	140.806	0.009
	CX	Cixi, Zhejiang	121.263°	30.369°	15	13	0.867	2.523	0.916	0.945	75.422*	0.006*
	QZ	Quanzhou, Fujian	118.667°	24.922°	14	4	0.286	0.895	0.459	0.586	3077.454*	0.337*
	FCG	Fangchenggang, Guangxi	108.213°	21.510°	15	1	0.070	0.000	0.000	NA	147.187*	0.037*
	Total	-	-	-	58	32	0.552	2.891	0.892	0.484	833.676*	0.044*

N is the number of samples, G is the number of MLGs, G/N is the number of MLGs divided by the number of individuals analyzed, H is the Shannon–Wiener index of diversity, λ is Simpson’s complement index of genotypic diversity, E is evenness, I_A_ is the index of association, r_d_ is the standardized index of association, and * indicates significant p-values (p = 0.001, permutations = 1,000).

According to the manufacturer’s instructions, genomic DNA was extracted from the leaves using the EasyPure Plant Genomic DNA Kit (Beijing TransGen Biotech Co., Ltd., Beijing, China). Based on the published nrITS sequences of 145 species of *Spartina* available from GenBank,^1^ nrITS (KM010334 of *S. anglica* and KM010330 of *S. alterniflora*) was used to identify *S. anglica* and *S. alterniflora* (Peterson et al., 2014). We confirmed that the HLD and DD populations were *S. anglica* and the other four populations were all *S. alterniflora* through multiple sequence alignment and neighbor joining (NJ) tree construction with 1,000 bootstrap by MAFFT v. 7 (Katoh et al., 2019; Supplementary Figure 2).

### Sequencing and Genotyping

All RAD libraries were created using 200 ng of genomic DNA from samples of *Spartina*. This DNA was double-digested with *Eco*RI and *Mse* l (New England Biolab, Beverly, MA, United States) and ligated to two-end adapters, allowing the resulting amplified fragments to bind to Illumina flow cells and uniquely identify the individual. All libraries with insert sizes of 300–500 bp were isolated by gel extraction and sequenced on the Illumina XTen platform using 150 nt paired-end reads (Jierui Biotech, Guangzhou, China). A total of ∼260 GB of data was obtained from Illumina sequencing of 88 samples from six populations. The data for each sample were split and filtered using the *process_radtags* module of Stacks 2.3e (Catchen et al., 2011). We removed reads with low quality, missing the restriction site or linked with incorrect barcodes. We combined each end of the retained reads from each sample. Here, we set two as the minimum depth of coverage (m) required to create a stack and three as the maximum distance (in nucleotides) allowed between stacks (M) within an individual. We then used the *cstacks* module in Stacks to build the catalogs for all these individuals, with two as the maximum number of mismatches allowed between individuals (n). We finally used the *cstacks, ustacks*, and *populations* modules in Stacks to obtain SNPs. We obtained the loci using the following limits in the *populations* module: 0.01 as the minor allele frequency, 75% as the percentage of individuals sharing the locus within the population, and appearance of the locus in all populations. Linkage disequilibrium (LD) between each pair was tested using VCFTOOLS v.0.1.15 (Danecek et al., 2011), and one marker was excluded from each pair with R2 > 0.8. The LD-excluded VCF data were further divided into *S. anglica* and *S. alterniflora* subsets for subsequent analysis by Tassel 5 (Bradbury et al., 2007), and the three datasets were converted into file formats necessary for analyses using PLINK v.1.9 (Purcell et al., 2007) and PGDSPIDER 2.0.9.0 (Lischer and Excoffier, 2012).

### Environmental Data

To test for correlations between genetic and environmental variables of *Spartina* in China, we required environmental data at the sampling locations. We extracted the standard nineteen bioclimatic variables (Supplementary Table 1) from the WORLDCLIM dataset (Version 2; period 1970–2000) (Hijmans et al., 2005) for the set of georeferenced locations of available localities using DIVA-GIS software (Hijmans et al., 2004). The total organic nitrogen (TN) and total phosphorus (TP) of the soil samples were determined using the Kjeldahl method and the molybdenum-antimony antispectrophotometric method at Bowu Company, Beijing. Soil electrical conductivity (EC) was detected by a New Digital DDS-307 Conductivity-Salinity Meter Tester in a mixture of 1:5 soil:distilled water as an index of soil salinity (S). The average TN, TP, and S values of three samples and the standard nineteen bioclimatic variables at each site were all used as environmental variables (Supplementary Table 1).

### Genetic Diversity and Clone Detection

Genetic diversity indices including the percentage of polymorphic loci (*%poly*), average nucleotide diversity (π), average observed heterozygosity per locus (*Ho*), and average expected heterozygosity per locus (*He*) were calculated using *populations* in Stacks version 2.3 based on the raw dataset (Catchen et al., 2011). Then, we used analysis of variation (ANOVA) to test for a difference in genetic diversity between *S. anglica* and *S. alterniflora* using the R 4.1.2 function “aov” (Chambers et al., 2017). The Tukey’s multiple comparisons test (TukeyHSD) was used for multiple comparisons (Yandell, 2017).

We referred to several analysis results to evaluate the reproductive systems of six populations. First, we calculated the average Wright’s inbreeding coefficient (*F*_*IS*_) using *populations* in Stacks version 2.3. The inbreeding coefficient (*F*_*IS*_) was often used to quantify the partial asexual reproduction population (Stoeckel and Masson, 2014). Negative *F*_*IS*_ values may reflect excess heterozygosity associated with reproduction via clonality (Pantoja et al., 2017).

Second, the genetic clones of *S. anglica* and *S. alterniflora* subsets were calculated using the package “poppr v. 2.9.3” based on the putatively neutral dataset (Kamvar et al., 2014, 2015), which can identify multilocus genotypes (MLGs) in clonal, partially clonal, and/or sexual reproduction populations. Clonal membership to the same genet can be inferred through a shared MLG among multiple individuals within a population. The true number of MLGs was determined by the “mlg.filter” function with a Nei’s distance (Nei, 1978) threshold determined using the cutoff_predictor tool, which finds a gap in the distance distribution. We investigated additional evidence of clonal reproduction through the “poppr” function (Kamvar et al., 2014, 2015). Since this function cannot work with a large dataset, we used two stricter datasets that restricted data analysis to one random SNP per locus (8,314 SNPs for *S. anglica* and 21,029 SNPs for *S. alterniflora*) to calculate the index of association (*I*_*A*_), the standardized index of association (*r*_*d*_), and their *p*-values (Supplementary Table 2). We also ensured that the MLGs obtained from strict datasets were consistent with the MLGs obtained from non-strict datasets. The *I*_*A*_ index determines individual’s recombination, which indicates panmixia, using the ratio of observed-to-expected variance in the number of loci (Agapow and Burt, 2001). The *r*_*d*_ index is a modification of *I*_*A*_ that removes sample size bias (Agapow and Burt, 2001). Clone correction was allowed, and the *p*-values of *I*_*A*_ and *r*_*d*_ were obtained with 999 permutations. We also constructed an unweighted pair group method with arithmetic mean (UPGMA) tree of all individuals, and minimum spanning networks (MSNs) of each MLG based on distance matrices.

We used “poppr” to calculate the clonal diversity of each population, including the Shannon–Wiener index (*H*) (Shannon, 1948), Simpson’s index (λ) (Simpson, 1949), and evenness (*E*). The parameter *G*/*N* was also estimated, and while it could be biased, it is frequently used (Saleh et al., 2012; Pantoja et al., 2017; Tsujimoto et al., 2020). *G* represents the number of MLGs and *N* is the number of samples.

### Population Structure

To infer the genetic structure of *Spartina* populations, we used ADMIXTURE version 1.3.0 (Alexander et al., 2009; Zhou et al., 2011) to estimate individual ancestries with the combined datasets of the two species and subsets of each species. We designated *K* = 1 to *K* = 9 as ancestral modes, and the most likely number of *K* given the populations was estimated according to the lowest cross-validation error (Alexander et al., 2009). We created an output file for the most likely number of *K* and visualized the results in the R environment. Discriminant analysis of principal components (DAPC) (Jombart et al., 2010) was performed using the R package “adegenet.” This method is more appropriate than others for the analysis of invasive species because it does not make any assumptions about migration-drift equilibrium (Jombart et al., 2010). The number of clusters was assessed for both species and their combined dataset, ranging from 1 to 10. The optimal number of clusters was identified based on the Bayesian information criterion (BIC), as suggested by Jombart et al. (2010). We used the function *find.*clusters to transform the original data into principal components (PCs), retaining 100 PCs in the analysis. Discriminant analysis was performed using 50 PCs (>90% of variance explained), and one, four or five eigenvalues were retained and examined. The optimal number of PCs to be retained in the final analysis was identified by the *optim.a.score* function with twenty independent runs.

Moreover, a redundancy analysis (RDA) within the “vegan” package (v.2.5–7) (Oksanen et al., 2021) in R 4.1.2 was used to assess whether environmental variables influenced the genetic differentiation of *S. alterniflora*. A principal component analysis (PCA) was performed by TASSEL 5 (Glaubitz et al., 2014). Variance inflation factors (VIF) were excluded through the “vif.cca” function of “vegan” package (Oksanen et al., 2021). The important environmental variables were chosen by the “forward.sel” function of the “adespatial” package (Dray et al., 2018). Then we used the “rdacca.hp” package to estimate the percentage contribution of explained variation to each or the total important environmental variables among the genetic groups, and the significance of the RDA results was tested with a global permutation (999 permutations) (Lai et al., 2022).

### Outlier Detection

The outlier loci were detected using two population differentiation (PD) analysis methods and two environment association (EA) analyses. The first PD method was implemented in ARLEQUIN (Excoffier et al., 2005), which identifies outliers by comparing genetic diversity and differentiation between populations. Both infinite island and hierarchical demographic models of this approach were used to estimate outliers, and we only chose the outliers identified by both models. The second PD method we used was in the “PCAdapt” package in R 4.1.2, which detects outlier loci based on principal component analysis (PCA). This approach overcomes the limitation of the traditional “outlier test” approach, which assumes an unrealistic island model of migration between populations. SNP with *qvalue* less than 0.01 was considered as an outlier (Capblancq et al., 2018).

For EA analysis, BAYENV identified outliers based on a correlation matrix of loci using SNPs, and 22 environmental variables and two geographical variables (longitude and latitude). A batch file estimated the BayesFactor with 100,000 MCMC steps. Regarding interpretation (Jeffreys, 1961), a BayesFactor more than 10 indicated strong supportive evidence for an association between an environmental parameter and a locus (Cullingham et al., 2014). Second, we used latent factor mixed models (LFMMs) implemented in the LEA package in R 4.1.2 (Frichot and François, 2015) to detect outliers correlated with climatic variables. The implementation of the LFMM is based on least-squares estimates (Caye et al., 2019). The prcomp function determines the number of latent factors (*K*). A fitted model was then used to perform association testing based on the ridge_lfmm function after ridge estimation. Finally, any obtained *p*-values were transformed into *qvalues*. The *qvalues* less than a cutoff of 0.01 were indicative of candidate loci. A Benjamini and Hochberg (1995) false discovery rate (FDR) correction of 5% was applied to *p*-values using the *qvalue* package in R (Storey et al., 2015). Binomial tests were used to assess differences in the number of outliers in the two species obtained by the four methods.

### Single Nucleotide Polymorphism Annotation

To gain insights into the potential adaptive significance of outlier loci, we obtained the flanking sequence of outlier loci identified by at least two methods from the RAD library. Functional annotation was performed using the online Basic Local Alignment Search Tool (BLAST) database. We searched the Genbank database using BLASTn with >60% sequence similarity. If the sequence was related to a protein, we further searched for its molecular function from UniProt^2^ and identified its related functions in plants according to literature. Then, we calculated correlations between allele frequencies of outlier loci and climate variables to identify putative genes associated with local adaptation.

## Results

### Genome Sequencing

A total of 1680,866,670 paired-end (PE) reads (read length = 150 bp, 252.13 Gbp) were obtained from the ddRAD library constructed for 30 individuals of *S. anglica* and 58 individuals of *S. alterniflora*. We obtained a total of 1674,211,878 reads (read length = 135 bp, 226.02 Gbp) after demultiplexing and removing reads of low quality. The number of reads for the samples varied from 3,632,316 to 52,525,600, with the average number of reads per sample being 19,025,135. Our analysis with ustacks showed that the number of ddRAD tags recovered from these samples varied from 248,767 to 1,385,706. Using cstacks, we obtained 4,664,866 loci from the 30 *S. anglica* and 58 *S. alterniflora* samples. After the initial filtering steps, 212,939 SNPs were retained, followed by removing sites showing linkage disequilibrium, and 169,522 SNPs were retained for all samples. Based on the dataset with 169,522 SNPs, a total of 90,671 SNPs were identified in the *S. alterniflora* subset, and 10,542 SNPs were identified in the *S. anglica* subset, with a minimum allele frequency of 0.01 (Supplementary Table 2).

### Mode of Reproduction

Using the clonal threshold of 0.124 identified by *cutoff_predictor*, we found 32 MLGs among 58 individuals of *S. alterniflora* (Supplementary Figure 3B), while 21 MLGs among 30 individuals of *S. anglica* were found with a threshold of 0.163 (Table 1 and Supplementary Figure 3D). Table 1 also shows genotypic (MLG) diversity calculated separately for each population. The FCG population of *S. alterniflora* had the lowest genotypic diversity, with a single MLG identified among 15 sampled individuals (G:N = 0.067). Other populations with low genotypic diversity were QZ (*S. alterniflora*) and HLD (*S. anglica*), which had G:N ratios of 28.6 and 40%, respectively. In contrast, populations DD (*S. anglica*) and TG (*S. alterniflora*) had G:N ratios of 100% (Table 1).

**FIGURE 1:**
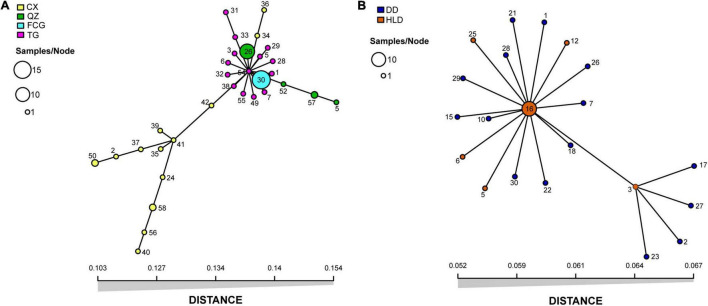
Minimum spanning networks (MSNs) of unique multilocus genotypes (MLGs) in populations of *Spartina alterniflora* **(A)** and *Spartina anglica* **(B)** collected from six fields in China. Each node represents a multilocus genotype (MLG), with size depending on the number of individuals with the MLG. The distance between the nodes represents the genetic distance between MLGs. Sampled fields are denoted by color, and the size of sample nodes represents either one or two isolates.

Both indices of association, *I*_*A*_ and *r*_*d*_, demonstrated that the population of genotypes was significantly different (*p* = 0.001) from the expected products of panmixia for populations HLD (*S. anglica*), CX, QZ, and FCG (*S. alterniflora*) (Table 1 and Supplementary Figure 4), indicating clonal reproduction as the prevalent mode of reproduction at these sites. The *p*-values of *I*_*A*_ and *r*_*d*_ were not significant (*p* = 1) only for populations DD (*S. anglica*) and TG (*S. alterniflora*) (Table 1 and Supplementary Figure 4), indicating that the null hypothesis of sexual reproduction could not be rejected for these populations. The inbreeding coefficients (*F*_*IS*_) of all *S. anglica* populations (DD and HLD) and the QZ and FCG populations of *S. alterniflora* were negative, showing a clear trend of heterozygote excess (Table 2). In addition, the minimum spanning networks (MSNs) based on Euclidean distances of the clone-corrected datasets showed no sign of reticulation (Figure 1). MLG 54 at the center of the *S. alterniflora* network consisted of only one individual from the TG population, and MLGs 26 and 30, which consisted of 10 individuals from the TG population and all individuals from the FCG population, were near the center (Figure 1A). Ten individuals of HLD belonging to MLG 16 were at the center of the *S. anglica* network, and other MLGs were found in only one individual (Figure 1B).

**TABLE 2:** Diversity indices of each sampled population of *Spartina anglica* and *Spartina alterniflora*.

Species	Pop	*%poly*	π	*H*_*O*_	*He*	*F*_*IS*_	*P*_*A*_
*Spartina anglica*	DD	0.484	0.035	0.042	0.034	−0.010	0.049
	HLD	0.474	0.035	0.052	0.034	−0.031	0.049
*Spartina alterniflora*	TG	2.143	0.151	0.121	0.145	0.090	0.220
	CX	1.555	0.120	0.110	0.115	0.029	0.073
	QZ	1.179	0.096	0.113	0.093	−0.020	0.057
	FCG	0.667	0.068	0.114	0.066	−0.089	0.049

*%poly is the percentage of polymorphic loci, π is average nucleotide diversity, H_o_ is observed heterozygosity, H_e_ is expected heterozygosity, F_IS_ is Wright’s inbreeding coefficient, and P_A_ is the frequency of private alleles.*

### Genetic Diversity

**FIGURE 2:**
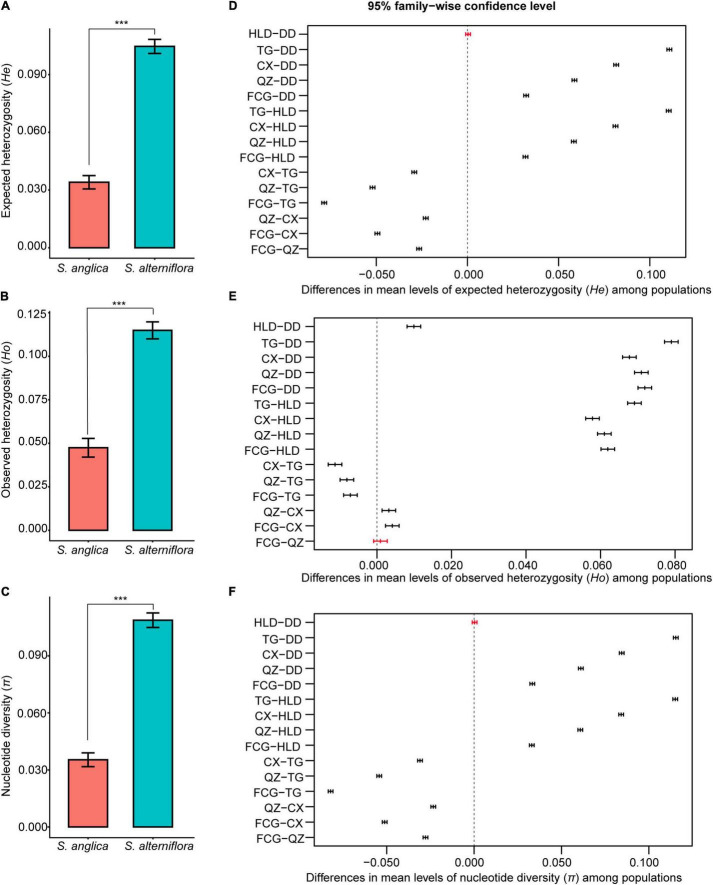
Comparison of expected heterozygosity (*He*), observed heterozygosity (*Ho*), and nucleotide diversity (π) between *Spartina anglica* and *S*. *alterniflora* **(A–C)** by the one-way ANOVA analysis, and among their populations **(D–F)** by Tukey’s multiple comparisons test. ***In panels **(A–C)** indicates *p* < 0.001. In panels **(D–F)**, the bars represent differences in mean levels of genetic diversity indicators (*He*, *Ho*, π) between populations, *p*-values of black bars were less than 0.001, while those of red bars were more than 0.05.

There was higher genetic diversity in *S. alterniflora* populations than in *S. anglica* populations in terms of nucleotide diversity (π, 0.035 vs. 0.068-0.151, *p* < 0.001), observed heterozygosity (*Ho*, 0.042–0.052 vs. 0.110–0.121, *p* < 0.001) and expected heterozygosity (*He*, 0.034 vs. 0.145-0.066, *p* < 0.001) (Table 2 and Figures 2A–C). TukeyHSD also revealed significant differences among the six populations (*p* < 0.001), except *He* and π between HLD and DD (*p* = 0.976, 0.988), *Ho* between FCG and QZ (*p* = 0.699, Figures 2D–F and Supplementary Table 3). The TG population had the highest frequency of private alleles (21.98%), while the DD and FCG populations had the lowest frequencies (4.85 and 4.91%, respectively) (Table 2).

### Genetic Structure

**FIGURE 3:**
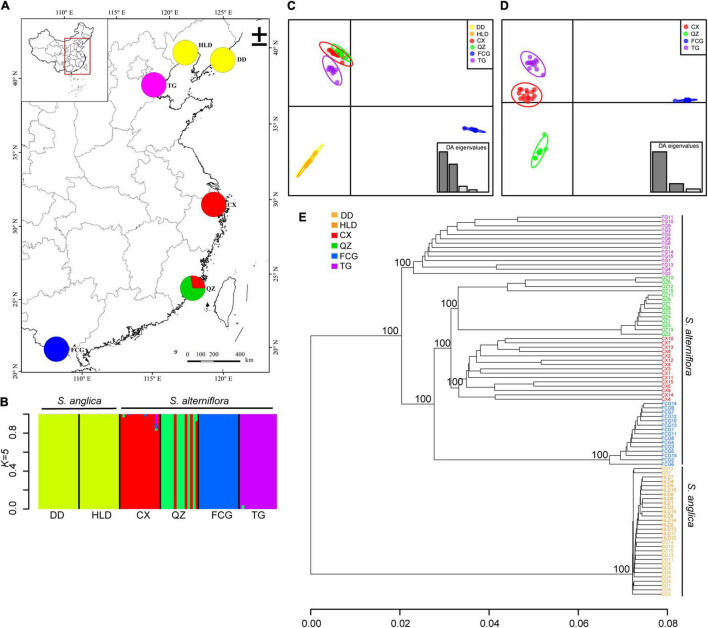
The genetic structure analysis of populations of *Spartina anglica* and *Spartina alterniflora* based on ADMIXTURE simulations, DAPC and UPGMA. **(A)** Map of collection sites for six populations. The pie chart shows the population cluster identified using ADMIXTURE when *K* = 5; **(B)** The ancestry composition of each individual of six populations. Each individual is represented by a vertical bar; **(C)** Scatter plots based on DAPC of all populations of *Spartina*, and **(D)** populations of *S. alterniflora*. Differently colored dots represent the individuals of different populations, and the insets indicate the eigenvalues from DAPC, with dark bars representing axes 1 and 2 of the plots. **(E)** Cluster analysis based on the genetic distance of six populations using a UPGMA tree with bootstrap support.

Five genetic clusters (*K* = 5) were identified using ADMIXTURE based on the lowest cross-validation error (Supplementary Figure 5). The two *Spartina* species were clearly separated (Figure 3). All populations of *S. alterniflora* were divided into four clusters, and only four individuals of QZ belonged to the red cluster (Figures 3A,B). The two populations of *S. anglica* did not separate from each other (Figures 3A,B). DAPC performed on SNP datasets showed clear genetic structure in *S. alterniflora* populations but not in *S. anglica* (Figure 3C and Supplementary Figures 5B–D). All four populations of *S. alterniflora* were also separated from each other, and individuals of the FCG population were most distanced from other populations’ individuals (Figure 3D and Supplementary Figure 5C). The two populations of *S. anglica* were not separated based on the subdataset (Supplementary Figure 5). A UPGMA tree showed that individuals of *S. alterniflora* and *S. anglica* were divided into two major clusters with high bootstrap support. Individuals of four populations of *S. alterniflora* also separated into four subclusters with 100% bootstrap support (Figure 3E). The genetic distances among *S. anglica* individuals did not show clear separation (Figure 3E).

Three important environmental variables were kept through forward selection in the RDA analysis, including the minimum temperature of the coldest month (BIO6), the mean temperature of the wettest quarter (BIO8), and the precipitation of the warmest quarter (BIO18), which were significantly associated with genetic variation among populations of *S. alterniflora* (*p* = 0.001). The total explained variation was 0.784, and the precipitation of the warmest quarter (BIO18; 38.58% explained variation, *p* = 0.001) was the most important predictor (Supplementary Figure 6 and Supplementary Table 4).

### Identification of Outliers and Analysis of Their Clines

A total of 7,868 and 238 outliers were identified in *S. alterniflora* and *S. anglica*, respectively, using four methods (Supplementary Figure 7). For the *S. alterniflora* subset, the two PD methods identified 4,791 outliers (4,145 from PCAdapt and 1,005 from ARLEQUIN), and the two EA methods identified 3,619 outliers (889 from BAYENV and 2,769 from LFMM). Only one outlier was identified by all four methods, 91 outliers were identified by three methods, and 848 outliers were identified by two methods (Supplementary Figure 7). For the *S. anglica* subset, the two PD methods identified 148 outliers (177 from PCAdapt and 93 from ARLEQUIN), and the two EA methods identified 31 outliers (12 from BAYENV and 19 from LFMM). None of the outliers were identified by all four methods, and only three outliers were identified by three methods; 60 outliers were identified by two methods (Supplementary Figure 7). The proportion of outliers obtained by the four methods was statistically different between the two species based on binomial tests (for PCAdapt, BAYENV and LFMM, *p* < 0.001; for ARLEQUIN, *p* < 0.05).

**FIGURE 4:**
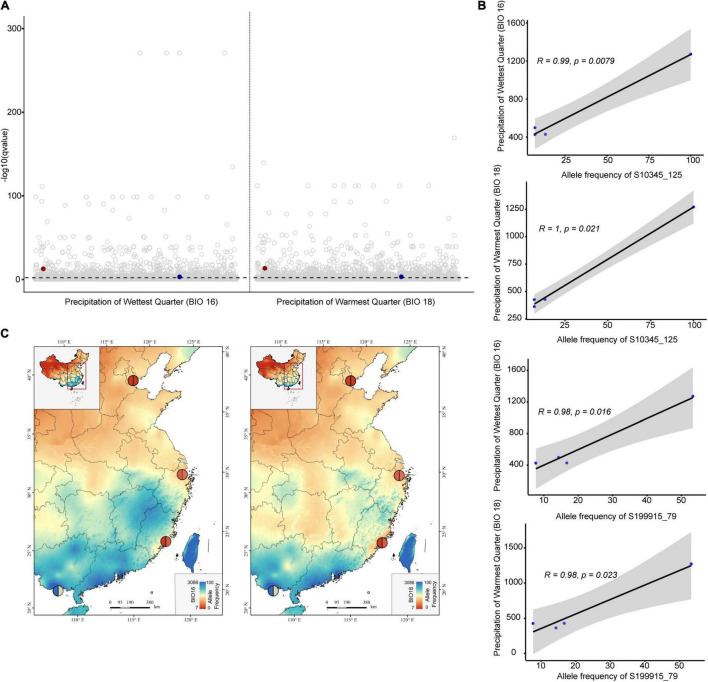
The outlier loci of *Spartina alterniflora* associated with climate variables based on the LFMM method. **(A)** Manhattan plots show the significance level for SNP associations with bioclimatic variables (BIO 16 and BIO18), the dark red points represent S10345_125, the dark blue points represent S199915_79, and the dashed line represents *qvalue* = 0.01. **(B,C)** Correlations between allele frequency and climate variables for S10345_125 and S199915_79 in populations of *S. alterniflora*. The colors of the pie chart on maps represent allele frequencies levels of S10345_125 (left half pie chart) and S199915_79 (right half pie chart).

Functional annotation was not successful for any outlier loci of *S. anglica*. Fifteen loci obtained from the *S. alterniflora* subset were annotated (Supplementary Table 5). All of them were identified by at least two methods (11 from ARLEQUIN, 13 from PCAdapt, five from LFMM, and one from BAYENV). Two-thirds of the annotated outlier loci (10 outlier loci) were obtained with the two PD methods, while only one was obtained with the two EA methods. We identified 12 adjacent genes with a range of functions that were associated with plant growth and development, and four genes were associated with abiotic stress and pathogen attack. Based on the LFMM method, two (S10345, S199915) and four (S10345, S107946, S125087, S190589) genes identified were associated with precipitation (BIO11, 12, 13, 16, 18) and temperature (BIO1, 3, 4, 6, 7, 8, 9), respectively (Supplementary Table 6). The locus S199915 was identified by both EA methods, and was associated with precipitation and the TN content by the LFMM method, while it was associated with precipitation and temperature by BAYENV method. However, we only found strong associations between precipitation (Bio13, Bio16, and Bio18) and allele frequencies of two SNPs (S10345_125 and S199915_79), respectively (*p* < 0.05, Figure 4 and Supplementary Figure 8).

## Discussion

Our study confirmed that there are two species of *Spartina* along the coast of China. We compared the genetic backgrounds of the two species that were intentionally introduced to China decades ago. We used genome wide SNPs to analyze differences in modes of reproduction, genetic diversity, and gene × environment correlations between the two species in an effort to explain the reasons behind their differential invasion success. These analyses point to that high genetic diversity and genetic adaptation in response to the environment might promote *S*. *alterniflora* spreading on the coast of China.

### Both High Clonality and Sexual Reproduction Were Revealed in the Two *Spartina* Species

Strong evidence of high clonal reproduction, such as a low number of genotypes, significant *I*_*A*_ and *r*_*d*_ (*p* < 0.001), and negative *F*_*IS*_, was found in populations of both *S. alterniflora* (FCG and QZ) and *S. anglica* (HLD). No reticulation of the minimum spanning networks (Figure 1) also provided an evidence of clonal spread (Maurice et al., 2019). However, sexual reproduction cannot be ignored in either species; population DD of *S. anglica* and population TG of *S. alterniflora* showed an extremely high G:N ratio. Although a slightly negative *F*_*IS*_ (−0.010) was found in population DD of *S. anglica*, we still suspected that sexual reproduction rather than clonality was the main mode of reproduction in this population in consideration of the high number of genotypes, non-significant *I*_*A*_ and *r*_*d*_, and lack of a large excess of heterozygotes. Negative *F*_*IS*_ was usually interpreted as a signal of clonal reproduction in previous studies (Balloux et al., 2003; Halkett et al., 2005), while it was recently considered unreliable for evaluating reproduction. Both non-negative and negative *F*_*IS*_ values can be frequently found in partially clonal populations, and the dynamics of *F*_*IS*_ in (partially) clonal populations might be impacted by genetic drift (Reichel et al., 2016). Therefore, we focused more on the number of genotypes and the indices of association (*I*_*A*_ and *r*_*d*_) when evaluating the mode of reproduction. Inconsistencies in *F*_*IS*_, *I*_*A*_, and *r*_*d*_ were also found in population CX, which might be a partially clonal population with significant indices of association but a non-negative *F*_*IS*_ (0.029). The results of reproduction analysis based on molecular genetic information in our study did not support the finding of previous studies that *S. anglica* mainly relies on asexual reproduction for population regeneration (Li et al., 2008). This may be due to population DD of *S. anglica* being located at a northern latitude (N 39.87°) with a lower annual mean temperature (9.3°C) and maximum temperature (28.88°C), while the population site in the study of Li et al. (2008) was located along the coast of the Yellow Sea in China (N 33.7°), with a higher annual mean temperature (15.0–15.6°C) and maximum temperature (39.0°C). The native distribution of *S. anglica* is in cool regions (the first record was in Lymington, Hampshire, England) (Gray and Raybould, 1991), and high temperatures might limit its seed production through less effective pollination or pollen germination (Li et al., 2008). Lower genotype diversities were found in populations FCG and QZ (latitudes below N 25°) than in TG and CX (latitudes above N 30°) (Table 1). This result was consistent with those of previous studies showing that sexual reproduction differed among populations of *S. alterniflora* in China and seed production decreased at low latitudes (Liu et al., 2016b).

High clonality is common in some notorious invasive alien species (Liu et al., 2006), such as *Pueraria montana* var. *lobata* (Bentley and Mauricio, 2016), *Eichhornia crassipes* (Barrett et al., 2008), *Pistia stratiotes*, and *Eichhornia crassipes* (Wang et al., 2016). In addition, most of these species are also capable of sexual propagation (Eckert, 2002) and plastic in their two methods of reproduction (Liu et al., 2006). Although low sexual reproduction may limit the local adaptation of alien species during an invasion due to little opportunity for genetic recombination, clonal reproduction can allow alien species to quickly establish populations regardless of whether the founder group sizes are small (Barrett et al., 2008). Our study revealed that the two *Spartina* species adopted both clonal and sexual reproduction strategies and might adjust seed production across latitudes. Therefore, the method of reproduction was not considered a major constraint on the dieback of *S. anglica* in China, even if this species may experience a reduced ability to produce seeds in low-latitude regions.

### The Effect of High Genetic Diversity on Invasion Cannot Be Ignored

The newly introduced species suffered from bottlenecks, followed by reduced fitness and evolutionary potential, yet they often become invasive, and this dilemma was called the genetic paradox of invasion (Roman and Darling, 2007; Estoup et al., 2016). The genetic paradox of invasions has been thoroughly discussed by researchers in recent decades (Frankham, 2005; Stapley et al., 2015; Estoup et al., 2016; Schrieber and Lachmuth, 2017), and its importance among genetic mechanisms of invasions may be overestimated (Estoup et al., 2016). Many invaders do not exhibit the genetic paradox phenomenon because they have no loss of genetic diversity and no significant adaptive challenge in the invaded area compared to their native areas (Bossdorf et al., 2005; Roman and Darling, 2007). Some studies have already found that a negative founder effect with low genetic diversity can be overcome by high phenotypic variability, which can help *Spartina* species establish well in new environments (Castillo et al., 2018; Li et al., 2019a). In this study, the average nucleotide diversity (π) and observed heterozygosity (*Ho*) of each population reflected lower genetic diversity of the failed invader *S. anglica* (π: 0.035, *Ho*: 0.042–0.052) relative to the successful invader *S. alterniflora*. (π: 0.068–0.151, *Ho*: 0.110–0.121). Even though population FCG had extremely low genotype diversity, its genetic diversity was significantly higher than that in populations of *S. anglica* (*p* < *0.001*, Figures 2D–F and Supplementary Table 3). This also suggests that differences in reproduction cannot explain the difference in genetic diversity observed here for *S. anglica* and *S. alterniflora*. Both species were intentionally introduced into China only once; the difference was that *S. anglica* came from a single source, while *S. alterniflora* came from multiple sources (Chung, 1993). The diverse genetic backgrounds resulting from multiple sources of *S. alterniflora* provided an opportunity for interbreeding and genetic recombination through sexual reproduction, whereas this was not possible for *S. anglica* with a single genetic source. Most previous studies also revealed that outcrossing of plants from the locations of origin (North Carolina, Georgia, and Florida in the US) of *S. alterniflora* generated a genetic admixture, and genetic variation remained high compared to those in the native populations (Bernik et al., 2016; Qiao et al., 2019; Xia et al., 2020). Moreover, only the TG and CX populations had higher expected heterozygosity (*He*, 0.145 and 0.115) than observed heterozygosity (*Ho*, 0.121 and 0.110), which may have been a reflection of earlier population bottlenecks (Rengefors et al., 2021), whereas, other populations did not show evidence of bottlenecks. Therefore, neither of the two *Spartina* species in China showed the genetic paradox of invasion. The low genetic diversity limiting population expansion of *S. anglica* might be due to inbreeding depression and loss of adaptive potential (Wellband et al., 2017, 2018). Significant differences in the levels of genetic diversity of successful and unsuccessful invasive species were consistent with those in a study of four aquatic invasive species (gobies and oysters) (Wellband et al., 2017). We also agree with the conclusion that the limitation of invasions via genetic diversity is species specific (Wellband et al., 2017).

Unlike most previous studies of *S. alterniflora* in China (Xia et al., 2015; Bernik et al., 2016; Qiao et al., 2019), ours revealed high genetic differentiation and low genetic admixture of populations based on structure analysis and DAPC, while the two populations of *S. anglica* in China could not be separated from each other. The findings of the current study do not support the previous findings of Wellband et al. (2017), who demonstrated greater divergence among populations of less-successful invasive species than among highly successful invasive species due to less gene flow among populations of the former. Population genetic structure in *S. alterniflora* in China was also found in the study of Xia et al. (2020), and they speculated that genetic divergence was mainly due to the low natural gene flow among populations. However, we believe that the existing genetic divergence is more likely a product of rapid adaptation, as Qiao et al. (2019) proved in their study. The significant association between environmental variables and genetic structure in this study also suggests that temperature (BIO6, 8) and precipitation (BIO18) are important drivers of genetic variation within *S. alterniflora* (Supplementary Figure 6). Therefore, high genetic variation promoted rapid genetic differentiation under environmental selection pressures is the possible explanation for why *S. alterniflora* in our study showed population genetic structure. Another reason is that RADseq technique afforded numerous SNPs with much more genetic information than other markers.

### Genomic Signature Provides Support for Local Adaptation of *Spartina alterniflora* in China

Rapid local adaptation of invasive alien species usually includes two aspects: phenotypic plasticity and genetic evolution (Wellband and Heath, 2017; VanWallendael et al., 2018; Cao et al., 2021). Early studies on rapid adaptive evolution were mostly based on phenotypic changes between generations through common garden experiments or reciprocal transplant experiments (Turner et al., 2014; Ochocki and Miller, 2017). Recently, large genome-wide variation provided a more thorough understanding of the relationship between adaptive evolution and climate at the gene level (Zenni et al., 2014; Bay et al., 2018; Wellband et al., 2018). Based on the genomic scan, the performance of outlier test methods has been evaluated and used to provide evidence for local adaptive evolution in several studies (Nielsen et al., 2018; Gallego-Garcia et al., 2019; Cao et al., 2021). For example, Bay et al. (2018) identified the genomic regions which adapted across contemporary climate gradients in populations of yellow warbler (*Setophaga petechia*) by LFMM. To reveal signatures of adaptation within two montane bumble bee species, Jackson et al. (2020) detect outliers through several EA methods, including LFMM, Bayenv2, and RDA. All of these methods could lead to false positives and negatives, and it is hard to identify which outlier loci are false positives (Lotterhos and Whitlock, 2014), We try to avoid missing true positive outliers and adopt a cautious screening attitude to avoid false positives, the same as previous studies did (Cullingham et al., 2014; Wellband et al., 2018; Cao et al., 2021).

Although cross-validation with multiple methods may increase the confidence of true positive outliers (Cullingham et al., 2014), we still consider the outlier loci, which can be successfully annotated and have significant correlations with environment variables, to be more reliable. As significant larger number of outliers in populations of *S. alterniflora* than in *S*. *anglica*, some of which were successfully annotated, provided evidence for rapid local adaptation in this study. It supports hypothesis of previous studies that rapid adaptive evolution is a key mechanism driving successful invasion of *S. alterniflora* along the coast of China through reciprocal transplant experiments (Qiao et al., 2019). Previous authors speculated that genetic admixture might promote the formation of super invasive genotypes with high competitive abilities (Qiao et al., 2019). We also cannot ignore the genetic admixture in our study; even though we chose four large geographically distant populations, genetic admixture was still found in population QZ. Therefore, a single introduction but with multiple sources could promote the adaptation of *S. alterniflora* to different environments in China even with the short time lag of invasion (Facon et al., 2006). Rapid adaptive evolution can promote phenotypic trait shifts to increase the fitness of alien species when they face new environments (Suarez and Tsutsui, 2008; Molina-Montenegro et al., 2018).

Previous studies revealed high phenotypic variation in *S. alterniflora* in China and proved the occurrence of latitudinal clines in vegetative and sexual traits of populations, especially a linear increase in seed set, which was the result of rapid evolution, through garden experiments at multiple latitudes (Liu et al., 2017, 2020). Moreover, some studies have also proven that seed traits are critical for dispersal syndromes and mechanisms allowing plants to cope with environmental stress (Baskin and Baskin, 1998; Moles et al., 2005), and adaptive evolution in an invasive plant can enhance the probability of seedling survival by increasing the thickness of the seed coat (Molina-Montenegro et al., 2018). Interestingly, the function of locus S50498 (Supplementary Table 5) in our study was related to grain size and shape in rice (Huang et al., 2017). In addition, we also found that locus S135470 (Supplementary Table 5) might play a crucial role in organelle biogenesis and seedling establishment (Rigas et al., 2009).

We further revealed two SNPs of locus S10345 and S199915 were strongly associated with precipitation variables. The highest allele frequencies at these SNPs both occurred in the Fangchengang city of Guangxi province, areas of the low latitude and high rainfall (Figure 4). The SNP on locus S10345 was related to diacylglycerol kinase 2 that belongs to the diacylglycerol kinase family (DGKs). DGKs phosphorylate diacylglycerol (DAG) to produce phosphatidic acid (PA), which is required for plant development and responses to both biotic and abiotic stress (Tan et al., 2018; Kue Foka et al., 2020), such as drought (Li et al., 2015), cold (Ruelland et al., 2002), and pathogen attack (Zhang and Xiao, 2015). The SNP on locus S199915 was the H + -pyrophosphatase gene, which is related to vacuolar membrane solute transport (Graus et al., 2018). Previous studies also proved up-regulation of this gene can enhance resistance to salt and drought stress in plants (Park et al., 2005; Brini et al., 2007). Nevertheless, there are clear limitations in this study to further confirm the role of these two genes in the adaptation of *S*. *alterniflora* to the environment, and we believe that these loci are worthy of further research and validation.

To conclude, we explored aspects of invasiveness from an evolutionary perspective through a genomic comparison of *S. alterniflora* and *S. anglica*, which have been introduced to China for several decades. Our results suggest that low sexual reproduction was not the reason for the dieback of *S. anglica* in coastal China. This further implies that the high genetic diversity caused by multiple introduction sources of *S. alterniflora* in China plays a role in the success of invasion. Moreover, comparisons of invading species and non-invasive related species might be more helpful for understanding the genetic paradox. Due to the limitations of the sampling strategy in our study (we only found two populations of *S. anglica* in China), we cannot directly demonstrate that rapid adaptive evolution was one of the main factors which promoted the success of *S*. *alterniflora* invasion. However, the evidence of the correlation between gene and environment of *S*. *alterniflora* revealed in this study might provide genetic support for previous research conclusion. Additionally, annotated outlier loci are worthwhile of further study in consideration that the functional protein-coding genes obtained from four populations of *S*. *alterniflora* might be critical for understanding processes influencing the range expansion of invasive species.

## Data Availability Statement

The data presented in the study are deposited in the GenBank repository, accession numbers SRR18493548-SRR18493635.

## Author Contributions

FL and CZ conceived the idea. FL, XL, JZ, and KG collected the samples in the field, performed the experiments, and collected the data. FL analyzed the data and wrote the original manuscript with edits completed by CZ. JL provided comments on the manuscript. All authors contributed to the final version before the manuscript was submitted.

## Conflict of Interest

The authors declare that the research was conducted in the absence of any commercial or financial relationships that could be construed as a potential conflict of interest.

## Publisher’s Note

All claims expressed in this article are solely those of the authors and do not necessarily represent those of their affiliated organizations, or those of the publisher, the editors and the reviewers. Any product that may be evaluated in this article, or claim that may be made by its manufacturer, is not guaranteed or endorsed by the publisher.

## Footnotes

1
www.ncbi.nlm.nih.gov/genbank

2
https://www.uniprot.org/
