# Supplementary material for: The Role of Genetic Factors in the Differential Invasion Success of Two Spartina Species in China
Source: Front Plant Sci. 2022 May 30;13:909429. doi: 10.3389/fpls.2022.909429 (PMC9196123; doi:10.3389/fpls.2022.909429)
Supplement: Supplementary file 1 [file Table_1.DOCX]

Supplementary Material

Supplementary Table 1 The standard nineteen bioclimatic variables and three soil physicochemical factors of six populations of *Spartina anglica* and *S. alterniflora*.

| Environmental Variables | *Spartina anglica* | | *S. alterniflora* | | | |
| --- | --- | --- | --- | --- | --- | --- |
|  | DD | HLD | CX | QZ | FCG | TG |
| BIO1 | 9.302 | 8.971 | 16.388 | 20.682 | 22.207 | 12.932 |
| BIO2 | 8.664 | 9.222 | 7.388 | 7.073 | 6.944 | 8.963 |
| BIO3 | 21.775 | 22.416 | 23.766 | 30.211 | 30.798 | 24.097 |
| BIO4 | 1110.374 | 1168.801 | 856.859 | 617.024 | 575.381 | 1044.11 |
| BIO5 | 28.883 | 29.126 | 32.588 | 32.296 | 32.768 | 30.612 |
| BIO6 | -10.904 | -12.013 | 1.5 | 8.884 | 10.22 | -6.584 |
| BIO7 | 39.787 | 41.139 | 31.088 | 23.412 | 22.548 | 37.196 |
| BIO8 | 22.107 | 22.538 | 26.699 | 22.802 | 28.345 | 25.043 |
| BIO9 | -5.148 | -6.22 | 8.051 | 18.876 | 14.394 | -0.645 |
| BIO10 | 22.107 | 22.538 | 26.699 | 27.902 | 28.345 | 25.043 |
| BIO11 | -5.148 | -6.22 | 5.723 | 13.119 | 14.394 | -0.645 |
| BIO12 | 939 | 560 | 1158 | 1152 | 2214 | 583 |
| BIO13 | 258 | 174 | 177 | 200 | 451 | 201 |
| BIO14 | 12 | 3 | 42 | 24 | 34 | 5 |
| BIO15 | 101.409 | 117.817 | 45.505 | 60.733 | 86.941 | 129.708 |
| BIO16 | 576 | 385 | 428 | 498 | 1272 | 426 |
| BIO17 | 42 | 10 | 141 | 83 | 123 | 19 |
| BIO18 | 576 | 385 | 428 | 363 | 1272 | 426 |
| BIO19 | 42 | 10 | 156 | 127 | 123 | 19 |
| TP | 505 | 449 | 688.515 | 514.65 | 73.942 | 521.756 |
| TN | 1655 | 1263 | 1012.241 | 1208.79 | 1107.871 | 1224.98 |
| S | 1.853 | 1.788 | 0.484 | 0.9 | 0.666 | 2.96 |

BIO1 = Annual Mean Temperature; BIO 2 = Mean Diurnal Range (Mean of monthly (max temp - min temp)); BIO 3 = Isothermality (bio2/bio7) (×100); BIO 4 = Temperature Seasonality (standard deviation ×100); BIO 5 = Max Temperature of Warmest Month; BIO 6 = Min Temperature of Coldest Month; BI0 7 = Temperature Annual Range (BIO5-BIO6); BIO 8 = Mean Temperature of Wettest Quarter; BI0 9 = Mean Temperature of Driest Quarter; BIO 10 = Mean Temperature of Warmest Quarter; BIO 11 = Mean Temperature of Coldest Quarter; BIO 12 = Annual Precipitation; BIO 13 = Precipitation of Wettest Month; BIO 14 = Precipitation of Driest Month; BIO 15 = Precipitation Seasonality (Coefficient of Variation); BIO 16 = Precipitation of Wettest Quarter; BIO 17 = Precipitation of Driest Quarter; BIO 18 = Precipitation of Warmest Quarter; BIO 19 = Precipitation of Coldest Quarter; TP = Soil total phosphorus; TN = Soil total organic nitrogen; S = Soil salinity.

Supplementary Table 2 The number of single nucleotide polymorphisms (SNPs) was identified at each step and used in different analyses.

| Dataset | Number of SNPs | Usage |
| --- | --- | --- |
| Raw dataset | 212,939 for each population | Genetic diversity, ANOVA analysis, and the average Wright’s inbreeding coefficient (*F_IS_*). |
| Putative neutral dataset | 169,522 for each population | Genetic structure analysis, discriminant analysis of principal components (DAPC), and unweighted pair group method with arithmetic mean (UPGMA) tree. |
| Subset datasets | 90,671 for *Spartina anglica* | Genetic clone analysis, clonal diversity analysis, and outlier detection for both species. And a redundancy analysis (RDA) only for *S. alterniflora.* |
|  | 10,542 for *S. alterniflora* |  |
| Strict datasets | 8,314 for *S. anglica* | The index of association (*I_A_*), the standardized index of association (*r_d_*) and their *p* values for both species. |
|  | 21,029 for *S. alterniflora* |  |

**Supplementary Table** 3 Pairwise comparisons of genetic diversity among six *Spartina* populations used TukeyHSD.

|  | *He* | | | | *Ho* | | | | *π* | | | |
| --- | --- | --- | --- | --- | --- | --- | --- | --- | --- | --- | --- | --- |
|  | *diff* | *lwr* | *upr* | *p* | *diff* | *lwr* | *upr* | *p* | *diff* | *lwr* | *upr* | *p* |
| HLD-DD | 0.0003 | -0.0010 | 0.0017 | 0.9762 | 0.0099 | 0.0081 | 0.0117 | 0.0000 | 0.0003 | -0.0011 | 0.0017 | 0.9883 |
| TG-DD | 0.1107 | 0.1094 | 0.1120 | 0.0000 | 0.0790 | 0.0772 | 0.0808 | 0.0000 | 0.1154 | 0.1140 | 0.1167 | 0.0000 |
| CX-DD | 0.0815 | 0.0802 | 0.0829 | 0.0000 | 0.0678 | 0.0659 | 0.0696 | 0.0000 | 0.0844 | 0.0831 | 0.0858 | 0.0000 |
| QZ-DD | 0.0587 | 0.0573 | 0.0600 | 0.0000 | 0.0709 | 0.0691 | 0.0728 | 0.0000 | 0.0611 | 0.0597 | 0.0624 | 0.0000 |
| FCG-DD | 0.0322 | 0.0308 | 0.0335 | 0.0000 | 0.0719 | 0.0700 | 0.0737 | 0.0000 | 0.0333 | 0.0319 | 0.0347 | 0.0000 |
| TG-HLD | 0.1104 | 0.1090 | 0.1117 | 0.0000 | 0.0691 | 0.0673 | 0.0709 | 0.0000 | 0.1151 | 0.1137 | 0.1164 | 0.0000 |
| CX-HLD | 0.0812 | 0.0799 | 0.0825 | 0.0000 | 0.0578 | 0.0560 | 0.0597 | 0.0000 | 0.0841 | 0.0828 | 0.0855 | 0.0000 |
| QZ-HLD | 0.0583 | 0.0570 | 0.0596 | 0.0000 | 0.0610 | 0.0592 | 0.0629 | 0.0000 | 0.0608 | 0.0594 | 0.0621 | 0.0000 |
| FCG-HLD | 0.0318 | 0.0305 | 0.0331 | 0.0000 | 0.0620 | 0.0601 | 0.0638 | 0.0000 | 0.0330 | 0.0316 | 0.0344 | 0.0000 |
| CX-TG | -0.0292 | -0.0305 | -0.0278 | 0.0000 | -0.0113 | -0.0131 | -0.0094 | 0.0000 | -0.0309 | -0.0323 | -0.0296 | 0.0000 |
| QZ-TG | -0.0520 | -0.0534 | -0.0507 | 0.0000 | -0.0081 | -0.0099 | -0.0062 | 0.0000 | -0.0543 | -0.0557 | -0.0529 | 0.0000 |
| FCG-TG | -0.0785 | -0.0799 | -0.0772 | 0.0000 | -0.0071 | -0.0090 | -0.0053 | 0.0000 | -0.0821 | -0.0835 | -0.0807 | 0.0000 |
| QZ-CX | -0.0229 | -0.0242 | -0.0216 | 0.0000 | 0.0032 | 0.0014 | 0.0050 | 0.0000 | -0.0234 | -0.0247 | -0.0220 | 0.0000 |
| FCG-CX | -0.0494 | -0.0507 | -0.0481 | 0.0000 | 0.0041 | 0.0023 | 0.0060 | 0.0000 | -0.0511 | -0.0525 | -0.0498 | 0.0000 |
| FCG-QZ | -0.0265 | -0.0278 | -0.0252 | 0.0000 | 0.0009 | -0.0009 | 0.0028 | 0.6992 | -0.0278 | -0.0292 | -0.0264 | 0.0000 |

*diff* = the difference in means; *lwr* and *upr* = confidence levels; *p* = the adjusted *p-values* for all possible pairs.

**Supplementary Table** 4 Redundancy analysis (RDA) results of *S*. *alterniflora* based on three important environmental variables.

| Environmental variables | Individual explained variation | I.perc(%) | Pr(>I) |
| --- | --- | --- | --- |
| BIO6 | 0.2390 | 30.47 | 0.001 *** |
| BIO8 | 0.2428 | 30.95 | 0.001 *** |
| BIO18 | 0.3026 | 38.58 | 0.001 *** |

BIO 6 = Min Temperature of Coldest Month; BIO 8 = Mean Temperature of Wettest Quarter; BIO 18 = Precipitation of Warmest Quarter; *** indicates significant *p* values.

**Supplementary Table** 5 Annotation of outlier SNPs of *Spartina alterniflora* obtained with at least two methods

| No. | Locus position | Methods | Gene ID from the NCBI database | Gene | Molecular function from UniProt | Function of Gene |
| --- | --- | --- | --- | --- | --- | --- |
| 1 | S2389 | PCAdapt, Arlequin | LOC112890662 | photosynthetic NDH subunit of lumenal location 4, chloroplastic | Peptidyl-prolyl cis-trans isomerase activity | Shown to be required for the stable accumulation and activity of NDH and related to photosynthesis (Ifuku et al., 2011). |
| 2 | S10345 | Arlequin, LFMM | LOC4351807 | diacylglycerol kinase 2, transcript variant X2, mRNA | diacylglycerol kinase activity | Generates phosphatidic acid (PA) (Gómez-Merino et al., 2004), which might be required for plant development and responses to abiotic stress and pathogen attack (Tan et al., 2018). Essential during gametogenesis and required for ER-localized phospholipid metabolism in vegetative and reproductive growth (Angkawijaya et al., 2020). |
| 3 | S50498 | PCAdapt, Arlequin | LOC123114474 | OVARIAN TUMOR DOMAIN-containing deubiquitinating enzyme 12-like, transcript variant X2, misc_RNA | Thiol-dependent deubiquitinase | Determines grain size and shape in rice (Huang et al., 2017) |
| 4 | S80823 | PCAdapt, Arlequin | LOC112889406 | DNA repair protein REV1, mRNA | Damaged DNA binding, deoxycytidyl transferase activity, DNA-directed DNA polymerase activity | Deoxycytidyl transferase involved in DNA repair. Impacts the aerial organ or root development of plants after UV irradiation (Santiago et al., 2008, Takahashi et al., 2005). |
| 5 | S84673 | PCAdapt, Arlequin |  | pentatricopeptide repeat-containing protein At1g80270, mitochondrial | mRNA binding | Involved in organellar RNA editing. Related to leaf size and morphogenesis (Doniwa et al., 2010). |
| 6 | S107946 | PCAdapt, LFMM | LOC112903577 | homeobox-leucine zipper protein ROC8-like, mRNA | DNA-binding transcription factor activity, RNA polymerase II-specific | Regulates the size of bulliform cells and lignin content in rice (Sun et al., 2020). |
| 7 | S123914 | PCAdapt, Arlequin | LOC117863867 | CHROMATIN REMODELING 19, transcript variant X9, mRNA | ATPase, acting on DNA | Acts as a transcriptional repressor and contributes to plant pathogen resistance (Kang et al., 2021). |
| 8 | S125087 | PCAdapt, LFMM | LOC120687647 | protein DROOPING LEAF-like, transcript variant X1, mRNA | Transcription cis-regulatory region binding | Plays a crucial role in specifying the carpel identity and floral meristem determinacy (Yamaguchi et al., 2004, Li et al., 2011). |
| 9 | S135470 | PCAdapt, Arlequin | LOC117843502 | lon protease homolog 2, peroxisomal, mRNA | ATP-dependent peptidase activity | Plays a crucial role in organelle biogenesis and seedling establishment (Rigas et al., 2009). |
| 10 | S135965 | PCAdapt, Arlequin | LOC120702797 | heparanase-like protein 3, mRNA | Beta-glucuronidase activity | Involved in posttranslational modifications of glycoproteins (Liu et al., 2018). Plays a role in the regulation of cell growth (Guerra-Guimarães et al., 2016). |
| 11 | S183676 | PCAdapt, Arlequin | LOC112899737 | trafficking protein particle complex II-specific subunit 120 homolog, mRNA | Specific subunit of the TRAPP II complex | The two conserved Arabidopsis TRAPPII subunits, CLUB/AtTRS130 and AtTRS120, have been shown to be required for cell plate formation (Jaber et al., 2010, Thellmann et al., 2010, Qi et al., 2011) with important functions in plant growth and development (Rybak et al., 2014) |
| 12 | S187301 | PCAdapt, Arlequin | LOC102707133 | vesicle-associated membrane protein 721, mRNA | Cell plate formation involved in plant-type cell wall biogenesis | Plays an essential role in plant growth and development (Yi et al., 2013). |
| 13 | S190589 | PCAdapt, LFMM | LOC112901471 | probable glycosyltransferase 2, mRNA | galactosyltransferase activity | Potential role in developmental and metabolic homeostasis (Lim and Bowles, 2004). |
| 14 | S199915 | LFMM Bayenv |  | H+-pyrophosphatase mRNA, complete cds | Catalyzes the specific cleavage of pyrophosphate | Related to salt- and drought-stress tolerance of plants (Graus et al., 2018). |
| 15 | S227664 | PCAdapt, Arlequin | LOC117840186 | isoleucine--tRNA ligase, chloroplastic/mitochondrial, mRNA | Aminoacyl-tRNA editing activity | Plays an essential role in plant gametogenesis and embryo development (Berg et al., 2005). |

ANGKAWIJAYA, A. E., NGUYEN, V. C., GUNAWAN, F. & NAKAMURA, Y. 2020. A pair of Arabidopsis diacylglycerol kinases essential for gametogenesis and endoplasmic reticulum phospholipid metabolism in leaves and flowers. *Plant Cell,* 32**,** 2602-2620.

BERG, M., ROGERS, R., MURALLA, R. & MEINKE, D. 2005. Requirement of aminoacyl‐tRNA synthetases for gametogenesis and embryo development in Arabidopsis. *The Plant Journal,* 44**,** 866-878.

DONIWA, Y., UEDA, M., UETA, M., WADA, A., KADOWAKI, K.-I. & TSUTSUMI, N. 2010. The involvement of a PPR protein of the P subfamily in partial RNA editing of an Arabidopsis mitochondrial transcript. *Gene,* 454**,** 39-46.

GóMEZ-MERINO, F. C., BREARLEY, C. A., ORNATOWSKA, M., ABDEL-HALIEM, M. E., ZANOR, M.-I. & MUELLER-ROEBER, B. 2004. AtDGK2, a novel diacylglycerol kinase from Arabidopsis thaliana, phosphorylates 1-stearoyl-2-arachidonoyl-sn-glycerol and 1, 2-dioleoyl-sn-glycerol and exhibits cold-inducible gene expression. *Journal of Biological Chemistry,* 279**,** 8230-8241.

GRAUS, D., KONRAD, K. R., BEMM, F., PATIR NEBIOGLU, M. G., LOREY, C., DUSCHA, K., GüTHOFF, T., HERRMANN, J., FERJANI, A. & CUIN, T. A. 2018. High V‐PPase activity is beneficial under high salt loads, but detrimental without salinity. *New Phytologist,* 219**,** 1421-1432.

GUERRA-GUIMARãES, L., PINHEIRO, C., CHAVES, I., BARROS, D. R. & RICARDO, C. P. 2016. Protein dynamics in the plant extracellular space. *Proteomes,* 4**,** 22.

HUANG, K., WANG, D., DUAN, P., ZHANG, B., XU, R., LI, N. & LI, Y. 2017. WIDE AND THICK GRAIN 1, which encodes an otubain‐like protease with deubiquitination activity, influences grain size and shape in rice. *The Plant Journal,* 91**,** 849-860.

IFUKU, K., ENDO, T., SHIKANAI, T. & ARO, E.-M. 2011. Structure of the Chloroplast NADH Dehydrogenase-Like Complex: Nomenclature for Nuclear-Encoded Subunits. *Plant and Cell Physiology,* 52**,** 1560-1568.

JABER, E., THIELE, K., KINDZIERSKI, V., LODERER, C., RYBAK, K., JüRGENS, G., MAYER, U., SöLLNER, R., WANNER, G. & ASSAAD, F. F. 2010. A putative TRAPPII tethering factor is required for cell plate assembly during cytokinesis in Arabidopsis. *New Phytologist,* 187**,** 751-763.

KANG, H., LIU, Y., FAN, T., MA, J., WU, D., HEITZ, T., SHEN, W.-H. & ZHU, Y. 2021. Arabidopsis CHROMATIN REMODELING 19 acts as a transcriptional repressor and contributes to plant pathogen resistance. *The Plant Cell*.

LI, H., LIANG, W., YIN, C., ZHU, L. & ZHANG, D. 2011. Genetic interaction of OsMADS3, DROOPING LEAF, and OsMADS13 in specifying rice floral organ identities and meristem determinacy. *Plant Physiology,* 156**,** 263-274.

LIM, E. K. & BOWLES, D. J. 2004. A class of plant glycosyltransferases involved in cellular homeostasis. *The EMBO journal,* 23**,** 2915-2922.

LIU, Y., CAO, D., MA, L., JIN, X., YANG, P., YE, F., LIU, P., GONG, Z. & WEI, C. 2018. TMT-based quantitative proteomics analysis reveals the response of tea plant (Camellia sinensis) to fluoride. *Journal of proteomics,* 176**,** 71-81.

QI, X., KANEDA, M., CHEN, J., GEITMANN, A. & ZHENG, H. 2011. A specific role for Arabidopsis TRAPPII in post‐Golgi trafficking that is crucial for cytokinesis and cell polarity. *The Plant Journal,* 68**,** 234-248.

RIGAS, S., DARAS, G., LAXA, M., MARATHIAS, N., FASSEAS, C., SWEETLOVE, L. J. & HATZOPOULOS, P. 2009. Role of Lon1 protease in post‐germinative growth and maintenance of mitochondrial function in Arabidopsis thaliana. *New Phytologist,* 181**,** 588-600.

RYBAK, K., STEINER, A., SYNEK, L., KLAEGER, S., KULICH, I., FACHER, E., WANNER, G., KUSTER, B., ZARSKY, V. & PERSSON, S. 2014. Plant cytokinesis is orchestrated by the sequential action of the TRAPPII and exocyst tethering complexes. *Developmental Cell,* 29**,** 607-620.

SANTIAGO, M. J., ALEJANDRE-DURáN, E., MUñOZ-SERRANO, A. & RUIZ-RUBIO, M. 2008. Two translesion synthesis DNA polymerase genes, AtPOLH and AtREV1, are involved in development and UV light resistance in Arabidopsis. *Journal of plant physiology,* 165**,** 1582-1591.

SUN, J., CUI, X., TENG, S., KUNNONG, Z., WANG, Y., CHEN, Z., SUN, X., WU, J., AI, P. & QUICK, W. P. 2020. HD‐ZIP IV gene Roc8 regulates the size of bulliform cells and lignin content in rice. *Plant biotechnology journal,* 18**,** 2559-2572.

TAKAHASHI, S., SAKAMOTO, A., SATO, S., KATO, T., TABATA, S. & TANAKA, A. 2005. Roles of Arabidopsis AtREV1 and AtREV7 in translesion synthesis. *Plant physiology,* 138**,** 870-881.

TAN, W.-J., YANG, Y.-C., ZHOU, Y., HUANG, L.-P., XU, L., CHEN, Q.-F., YU, L.-J. & XIAO, S. 2018. DIACYLGLYCEROL ACYLTRANSFERASE and DIACYLGLYCEROL KINASE modulate triacylglycerol and phosphatidic acid production in the plant response to freezing stress. *Plant physiology,* 177**,** 1303-1318.

THELLMANN, M., RYBAK, K., THIELE, K., WANNER, G. & ASSAAD, F. F. 2010. Tethering factors required for cytokinesis in Arabidopsis. *Plant physiology,* 154**,** 720-732.

YAMAGUCHI, T., NAGASAWA, N., KAWASAKI, S., MATSUOKA, M., NAGATO, Y. & HIRANO, H.-Y. 2004. The YABBY gene DROOPING LEAF regulates carpel specification and midrib development in Oryza sativa. *The Plant Cell,* 16**,** 500-509.

YI, C., PARK, S., YUN, H. S. & KWON, C. 2013. Vesicle-associated membrane proteins 721 and 722 are required for unimpeded growth of Arabidopsis under ABA application. *Journal of plant physiology,* 170**,** 529-533.

**Supplementary Table** 6 The -log_10_(*qvalue*) of annotated outlier SNP site at related environmental variables which obtained from LFMM and the BayesFactor which obtained from BAYENV.

| No. | Site position | Method | Environmental variables | -log_10_(*qvalue*)/ BayesFactor |
| --- | --- | --- | --- | --- |
| 1 | S10345_125 | LFMM | BIO8 | 3.182 |
|  |  | LFMM | BIO12 | 7.413 |
|  |  | LFMM | BIO13 | 13.590 |
|  |  | LFMM | BIO16 | 13.106 |
|  |  | LFMM | BIO18 | 13.731 |
|  |  | LFMM | TN | 10.991 |
| 2 | S10345_104 | LFMM | TN | 2.303 |
| 3 | S107946_124 | LFMM | BIO8 | 4.667 |
| 4 | S125087_23 | LFMM | BIO1 | 3.044 |
|  |  | LFMM | BIO3 | 5.149 |
|  |  | LFMM | BIO4 | 3.301 |
|  |  | LFMM | BIO6 | 2.725 |
|  |  | LFMM | BIO7 | 3.336 |
|  |  | LFMM | BIO9 | 3.418 |
|  |  | LFMM | BIO11 | 3.142 |
| 5 | S190589_7 | LFMM | BIO8 | 7.388 |
|  |  | LFMM | BIO9 | 3.269 |
| 6 | S199915_79 | LFMM | BIO13 | 3.190 |
|  |  | LFMM | BIO16 | 3.040 |
|  |  | LFMM | BIO18 | 3.163 |
|  |  | BAYENV | BIO1 | 23.542 |
|  |  | BAYENV | BIO3 | 38.768 |
|  |  | BAYENV | BIO5 | 21.849 |
|  |  | BAYENV | BIO6 | 40.917 |
|  |  | BAYENV | BIO8 | 12.456 |
|  |  | BAYENV | BIO9 | 39.844 |
|  |  | BAYENV | BIO10 | 41.745 |
|  |  | BAYENV | BIO11 | 33.868 |
|  |  | BAYENV | BIO13 | 51.015 |
|  |  | BAYENV | BIO15 | 54.601 |
|  |  | BAYENV | BIO18 | 30.379 |
|  |  | BAYENV | TP | 20.894 |
|  |  | BAYENV | S | 23.542 |

BIO1 = Annual Mean Temperature; BIO 3 = Isothermality (bio2/bio7) (×100); BIO 4 = Temperature Seasonality (standard deviation ×100); BIO 5 = Max Temperature of Warmest Month; BIO 6 = Min Temperature of Coldest Month; BI0 7 = Temperature Annual Range (BIO5-BIO6); BIO 8 = Mean Temperature of Wettest Quarter; BI0 9 = Mean Temperature of Driest Quarter; BIO 10 = Mean Temperature of Warmest Quarter; BIO 11 = Mean Temperature of Coldest Quarter; BIO 12 = Annual Precipitation; BIO 13 = Precipitation of Wettest Month; BIO 15 = Precipitation Seasonality (Coefficient of Variation); BIO 16 = Precipitation of Wettest Quarter; BIO 18 = Precipitation of Warmest Quarter; TP = Soil total phosphorus; TN = Soil total organic nitrogen; S = Soil salinity.


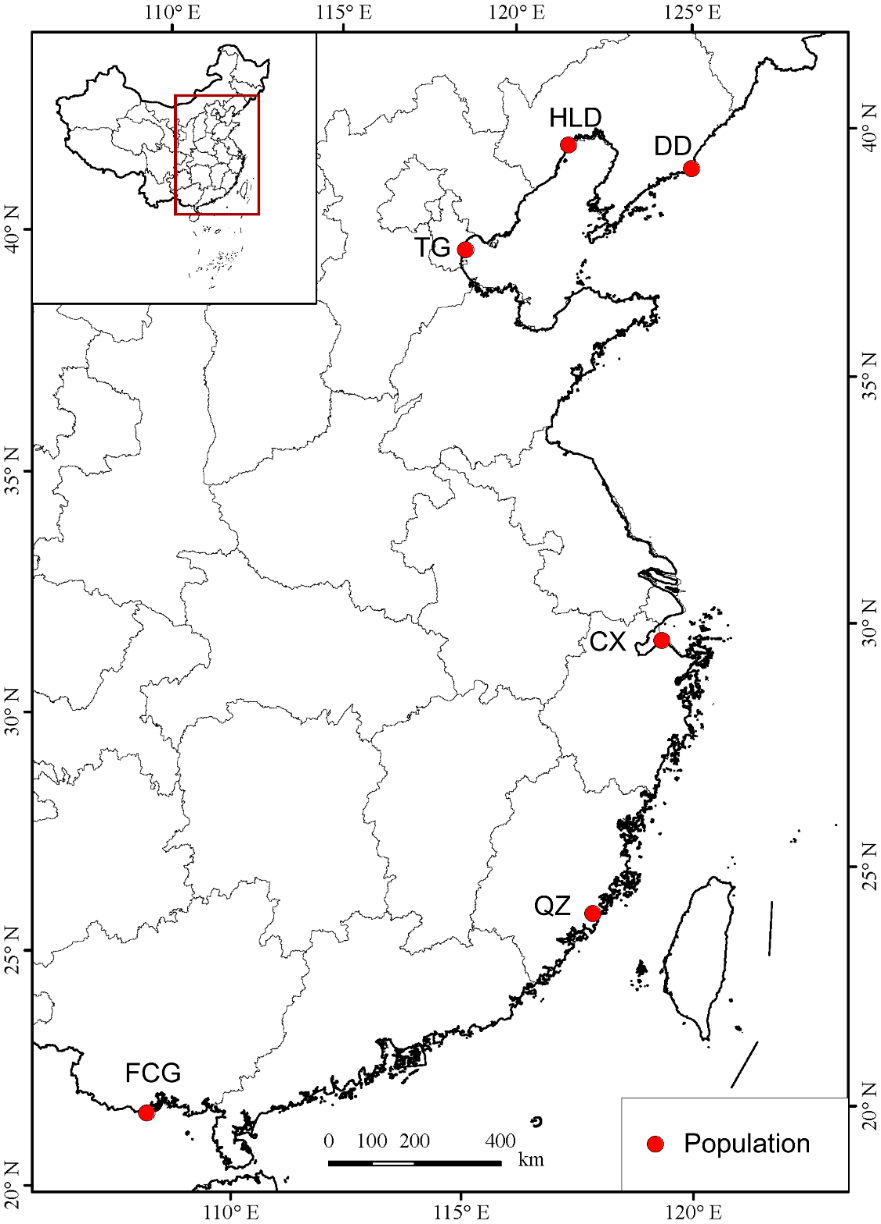


Supplementary Figure 1 The distributions of six populations of two *Spartina* species sampled in this study.


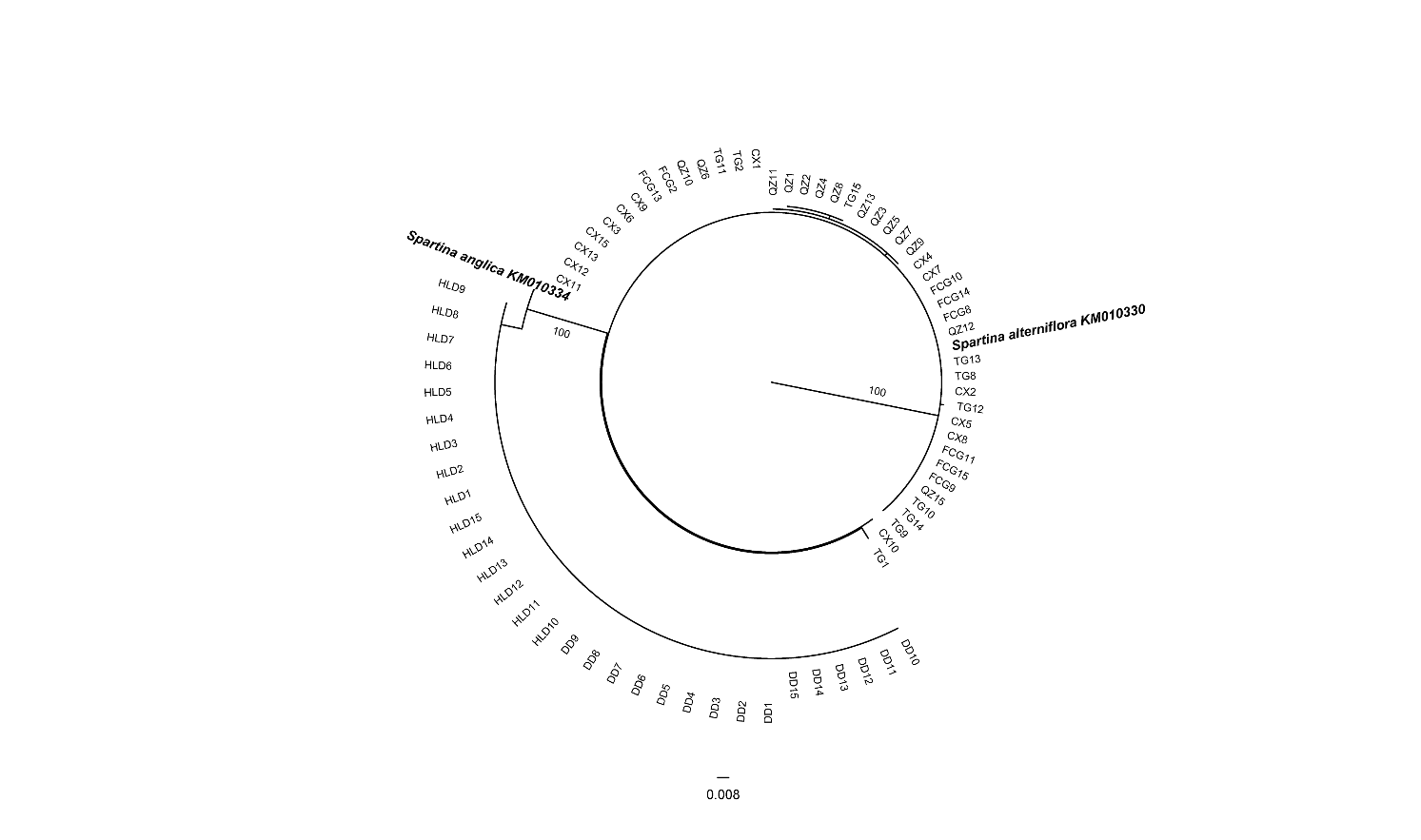


Supplementary Figure 2 The neighbor joining (NJ) tree based on nrITS sequences of two *Spartina* species in China, with two reference sequences from Genbank.


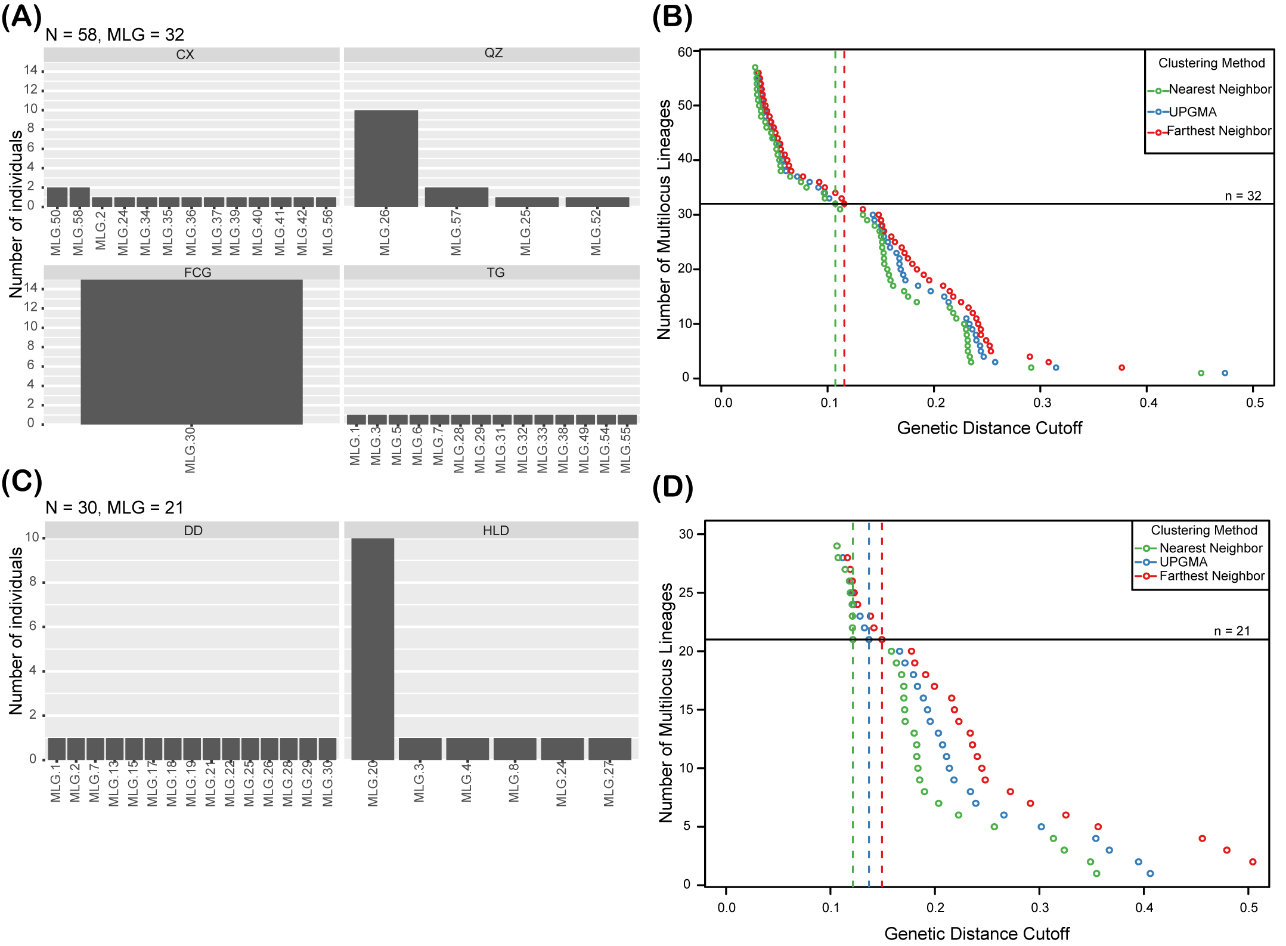


**Supplementary** Figure 3 Multilocus genotype histograms of the two *Spartina* species. (A) Distribution of 32 multilocus genotypes from *S*. *alterniflora* populations; (B) The clonal threshold identified by genetic distance cutoff based on *S*. *alterniflora* dataset; (C) Distribution of 21 multilocus genotypes from *S*. *anglica* populations; (D) The clonal threshold identified by genetic distance cutoff based on *S*. *anglica* dataset.


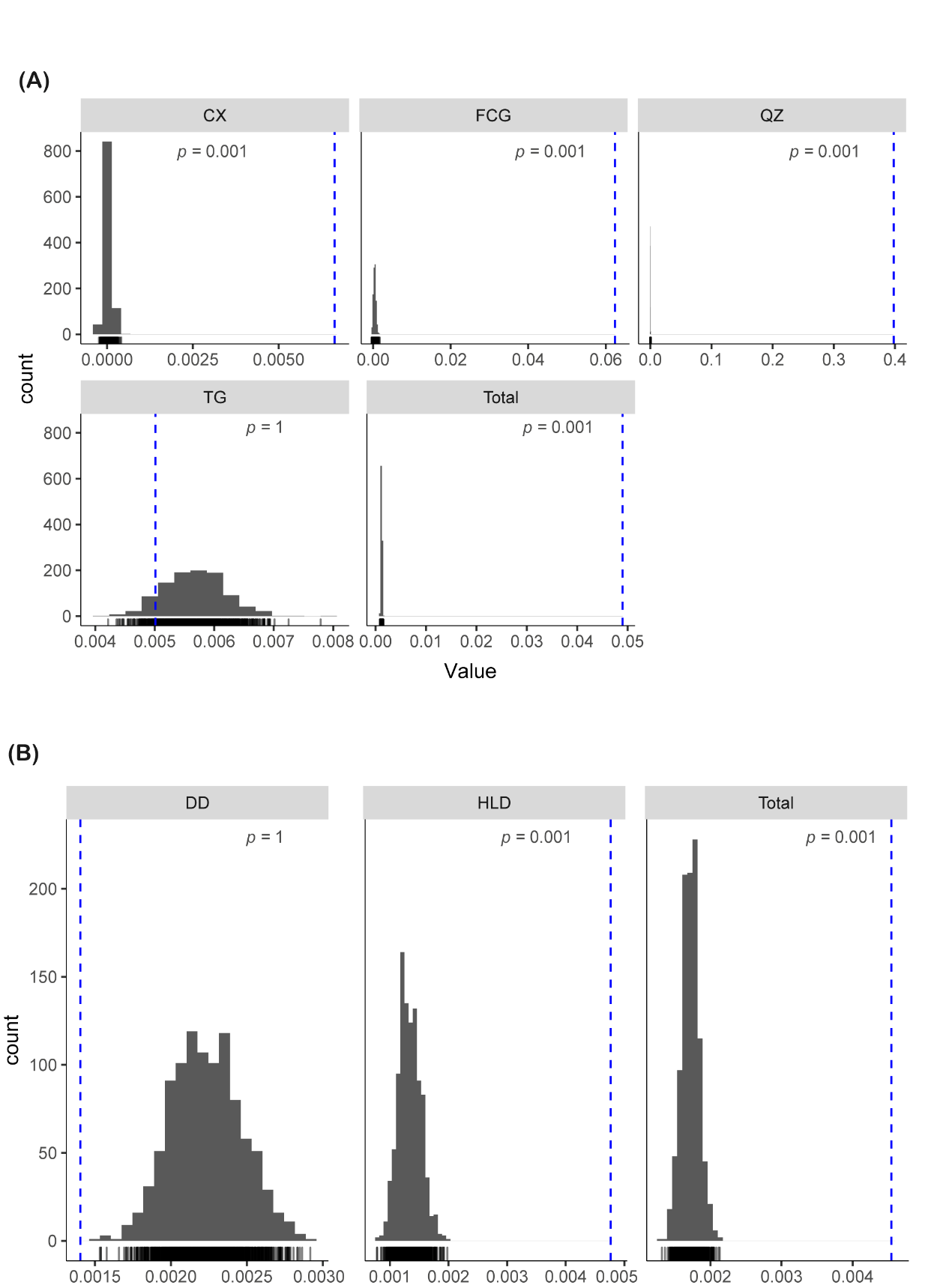


**Supplementary** Figure 4 Visualizations of tests for linkage disequilibrium of the two *Spartina* species. Blue dashed lines indicate observed values of *r_d_* compared to histograms showing results of 999 permutations. (A) *S. alterniflora* populations; (B) *S*. *anglica* populations.

**
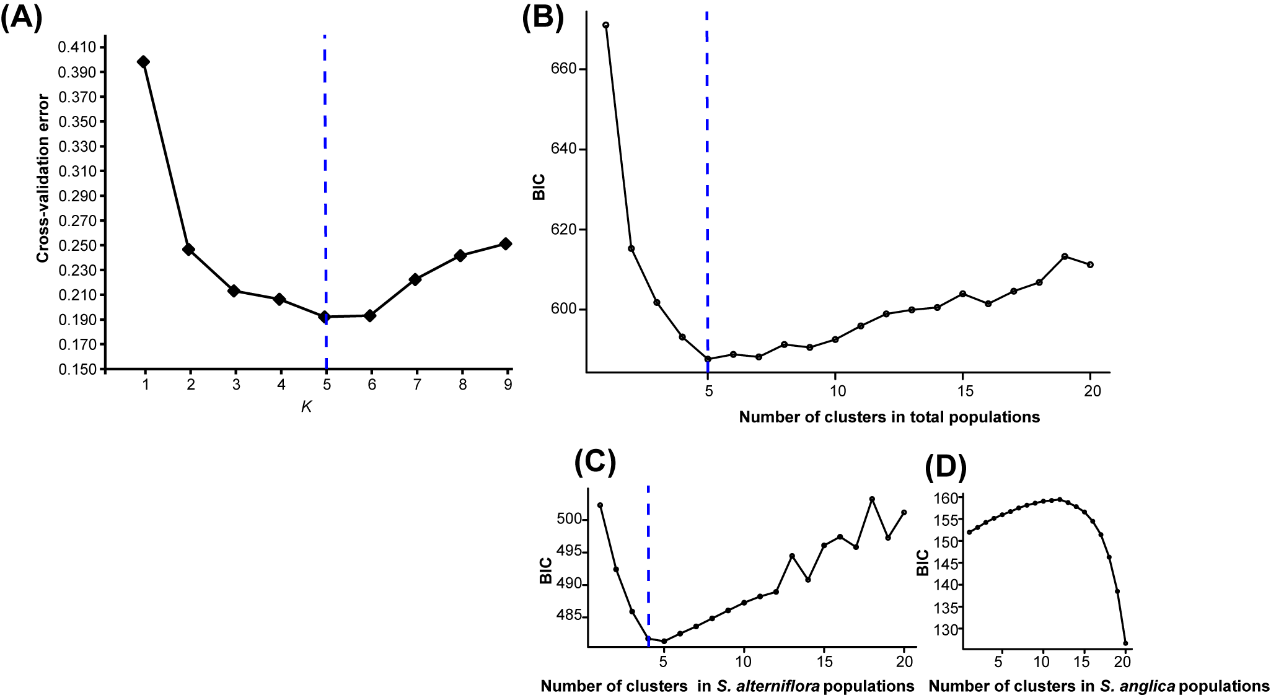
**

Supplementary Figure 5 Genetic structure analysis of *Spartina* in China. Blue dashed lines indicate the best *K*/clusters. (A) ADMIXTURE cross validation errors for each *K* value, and the best *K* exhibit the lowest cross validation error. (B-D) The BIC values of total popualtions (B), *S. alterniflora* (C) and *S. anglica* (D) for increasing values of *K* (number of clusters) based on DAPC analysis, and the best number of clusters locate at an elbow in the curve of BIC values.


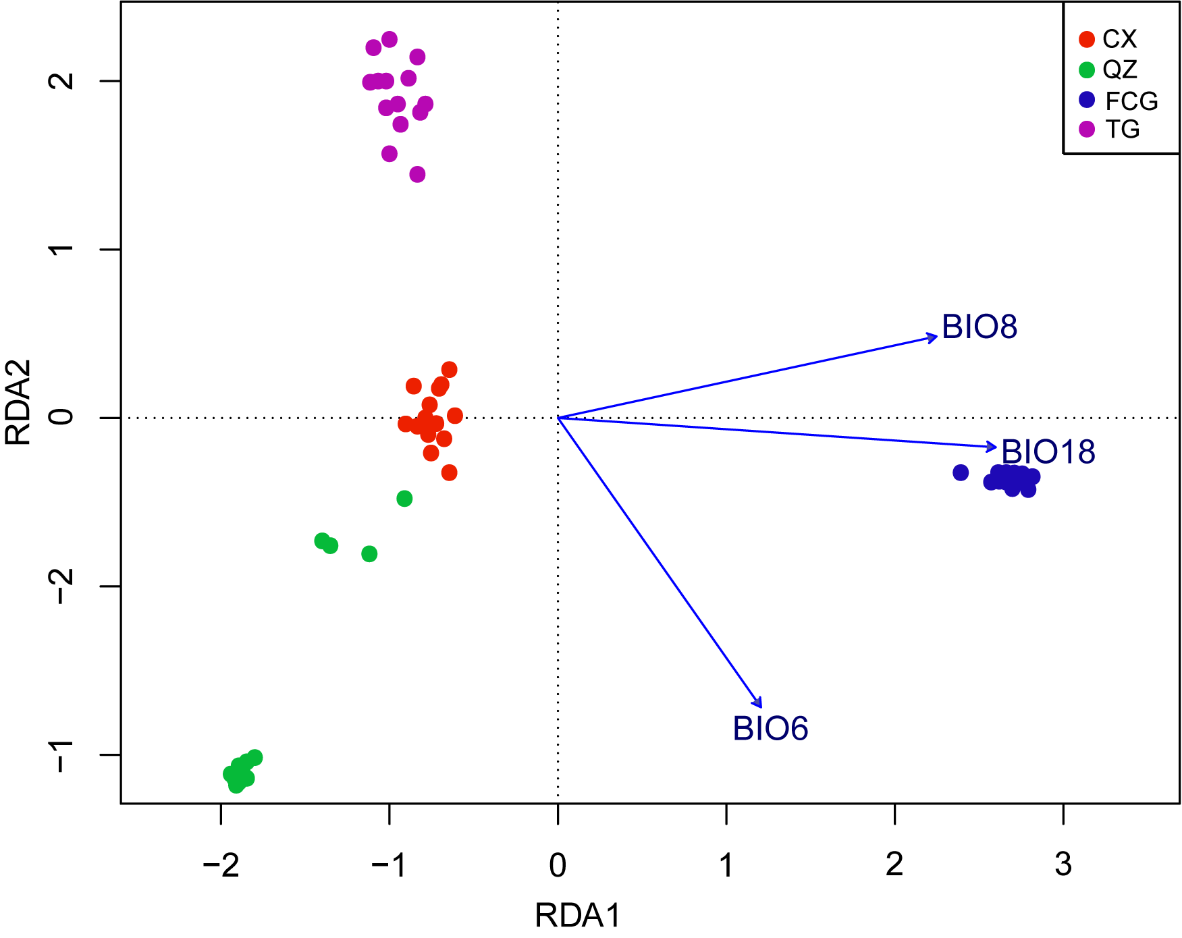


**Supplementary** Figure 6 Gene × environment association analyses. Colour points represent individual genotypes of six populations on the first two RDA axes. The arrows represent important environmental variables. BIO 6 = Min Temperature of Coldest Month; BIO 8 = Mean Temperature of Wettest Quarter; BIO 18 = Precipitation of Warmest Quarter.


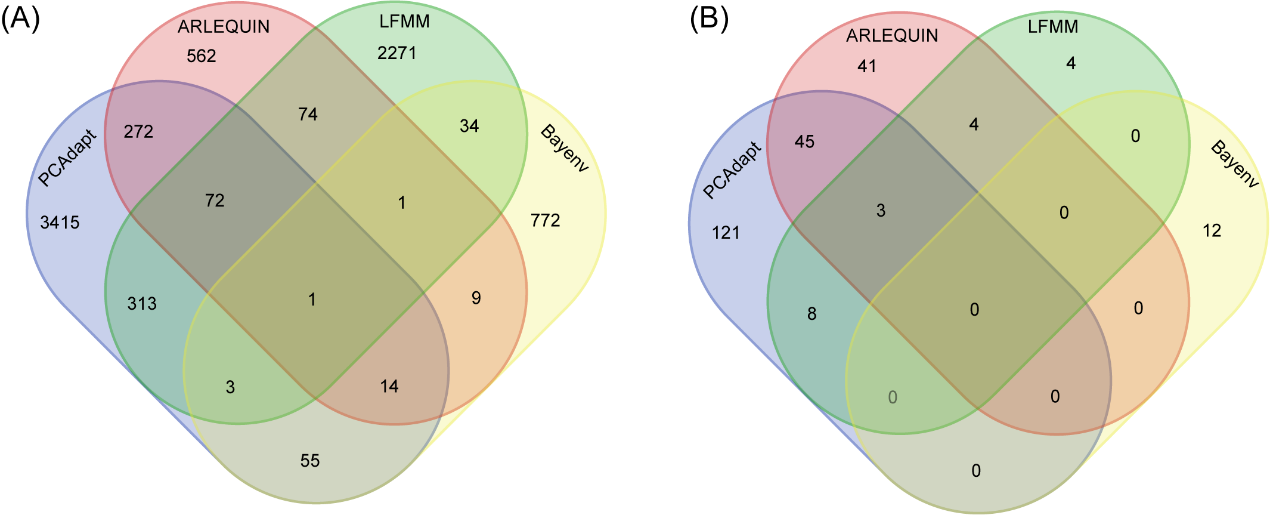


**Supplementary** Figure 7 Venn diagrams illustrating the overlap in outliers detected across the two datasets with the different outlier detection methods: (A) *Spartina alterniflora* subset, (B) *S. anglica* subset.


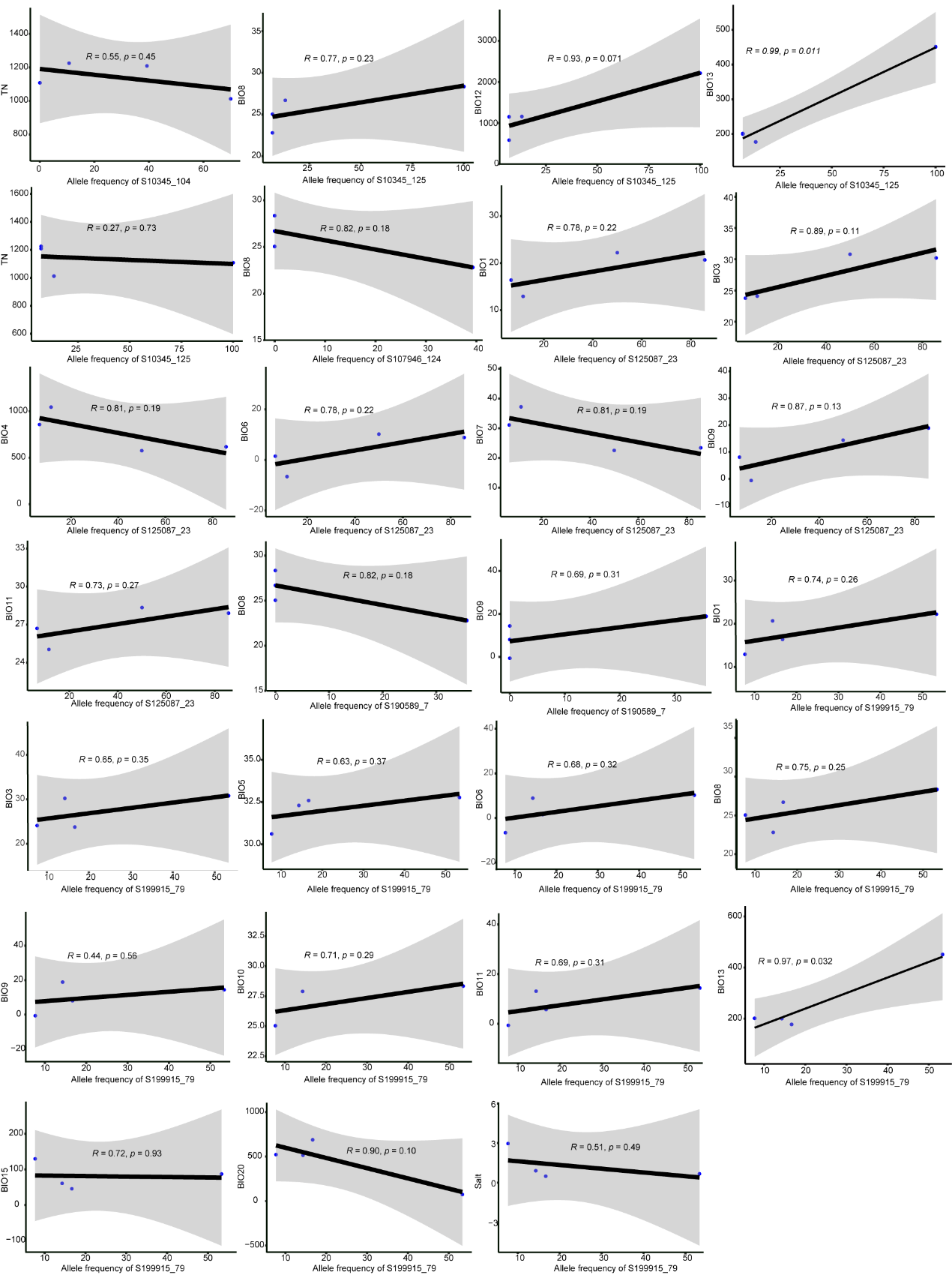


**Supplementary** Figure 8 Correlations between allele frequency and climate variables for annotated outlier SNP site obtained from LFMM and BAYENV.
